# Human walking biomechanics on sand substrates of varying foot sinking depth

**DOI:** 10.1242/jeb.246787

**Published:** 2024-11-05

**Authors:** Barbara F. Grant, James P. Charles, Kristiaan D'Août, Peter L. Falkingham, Karl T. Bates

**Affiliations:** ^1^ Department of Musculoskeletal and Ageing Science, Institute of Life Course and Medical Sciences, University of Liverpool, William Henry Duncan Building, 6 West Derby Street, Liverpool L7 8TX, UK; ^2^ School of Biological and Environmental Sciences, Liverpool John Moores University, James Parsons Building, Bryon Street, Liverpool L3 3AF, UK

**Keywords:** Kinematics, Locomotion, Compliant substrate, Gait, EMG

## Abstract

Our current understanding of human gait is mostly based on studies using hard, level surfaces in a laboratory environment. However, humans navigate a wide range of different substrates every day, which incur varied demands on stability and efficiency. Several studies have shown that when walking on natural compliant substrates there is an increase in energy expenditure. However, these studies report variable changes to other aspects of gait such as muscle activity. Discrepancies between studies exist even within substrate types (e.g. sand), which suggests that relatively ‘fine-scale’ differences in substrate properties exert quantifiable influences on gait mechanics. In this study, we compared human walking mechanics on a range of sand substrates that vary in overall foot sinking depth. We demonstrated that variation in the overall sinking depth in sand was associated with statistically significant changes in joint angles and spatiotemporal variables in human walking but exerted relatively little influence on pendular energy recovery and muscle activations. Significant correlated changes between gait metrics were frequently recovered, suggesting a degree of coupled or mechanistic interaction in their variation within and across substrates. However, only walking speed (and its associated spatiotemporal variables) correlated frequently with absolute foot sinkage depth within individual sand substrates, but not across them. This suggests that a causative relationship between walking speed and foot sinkage depth within individual sand substates is not coupled with systematic changes in joint kinematics and muscle activity in the same way as is observed across sand substrates.

## Teaser

**Summary:** In human walking, variation in the overall sinking depth in sand is associated with significant changes in gait variables such as joint angles and spatiotemporal variables.

## INTRODUCTION

In everyday life, humans and other animals navigate complex environments with heterogeneous terrain. Variations in substrate properties, such as compliance (e.g. foot sinking depth), impact how they walk across the surface to maintain manoeuvrability, grip and stability (Holowka et al., 2022; Peyré-Tartaruga and Coertjens, 2018). Previous studies have found that humans incur a much greater metabolic cost of locomotion when walking or running on natural, compliant substrates such as grass (Davies and Mackinnon, 2006; Pinnington and Dawson, 2001), snow (Pandolf et al., 1976) and sand (Davies and Mackinnon, 2006; Lejeune et al., 1998; Zamparo et al., 1992) compared with solid surfaces. The term ‘compliant’ has been used broadly within the field (Holowka et al., 2022; Kerdok et al., 2002; Lejeune et al., 1998; Pinnington and Dawson, 2001; Soule and Goldman, 1972; Zamparo et al., 1992) to refer to any substrate that has non-negligible deformation (elastic or plastic) under loads typically generated during human locomotion. However, the reported increases in energy expenditure vary not only between different types of compliant substrate but also between different studies using the same substrate type. During walking on sand, Davies and Mackinnon (2006) found that energy expenditure was up to 1.34 times greater, Zamparo et al. (1992) found an increase of up to 1.8 times and Lejeune et al. (1998) found an increase of 2.7 times compared with hard floor. A variety of different, and sometimes contradictory, reasons have been invoked to explain these increased energetic costs.

Pinnington and Dawson (2001) proposed that the differences in energetic costs between studies were likely due to variations in sand properties and/or methodology. Zamparo et al. (1992) proposed that the increase in energetic costs on sand could be attributed to a reduced recovery of potential and kinetic energy at each stride, based on calculations by Cavagna et al. (1976) which modelled the body as a simple inverted pendulum. However, more recent studies suggest that these mechanical energy exchange variables do not necessarily strongly correlate with the amount of mechanical energy exchange. Instead, mechanical work is predominately required to redirect the centre of mass (CoM) velocity vector during step-to-step transitions and collisional losses that occur during these transitions account for a considerable proportion of the metabolic cost of walking (Bertram and Hasaneini, 2013; Donelan et al., 2002; Kuo et al., 2005). Lejeune et al. (1998) attributed their increased energetic costs to increased mechanical work done on the sand and a decrease in the efficiency of positive work done by the muscles and tendons. Zamparo et al. (1992) and Pinnington and Dawson (2001) suggested that foot slippage during push-off contributes to increased energetic costs when walking on sand. During running on sand, participants displayed greater hip and knee flexion and greater co-activation of the knee and ankle muscles (Pinnington and Dawson, 2001; Pinnington et al., 2005), whereas Bates et al. (2013) suggested that there may be increased activation in ankle extensor muscles in the stance phase. Giatsis et al. (2004) observed an increased range of motion at the ankle joint prior to push-off during jumping on sand, which may have been caused by foot slippage, and Pinnington and Dawson (2001) suggested that on compliant surfaces, the foot is in contact with the ground for longer as a mechanism to improve stability and reduce foot slippage.

This variation in increases of metabolic cost of transport, and the gait variables associated with it, suggests that differences in mechanical properties and/or behaviour of sediment may exert quantifiable kinematic and kinetic responses. However, there is currently little understanding of how humans adapt their gait to different substrate properties, particularly for natural sediments such as sand that exhibit complex behaviour. A study by Pandolf et al. (1976) found a positive linear relationship between increasing footprint depth in snow and an increase in energetic costs during walking, suggesting there may be some causative link between the depth of depression into a compliant substrate and energy expenditure. Therefore, in the present study, we investigated how overall sinking depth (measured by the vertical position of markers on the foot) affects human gait kinematics and muscle activities during walking on sand. Based on previous work, we tested the predictions that on substrates with greater average foot sinking depth, (P1) pendular energy exchange mechanisms will incur reduced efficiency, (P2) stance time and stride width will increase and walking speed and stride length will decrease, (P3) there will be greater joint excursions at the hip, knee and ankle joints owing primarily to an increase in peak joint flexion and (P4) there will be greater muscle activation in the lower limb muscles. From these predicted differences between substrates, we derive two further mechanistic predictions: first, that (P5) changes in pendular energy exchange, muscle activity and spatiotemporal and joint kinematics will correlate with each other both within and across all substrates; and second, that (P6) that changes in these gait metrics will correlate with variation in absolute foot sinking depth within and across the sand substrates.

## MATERIALS AND METHODS

### Experimental data collection

**Fig. 1.**
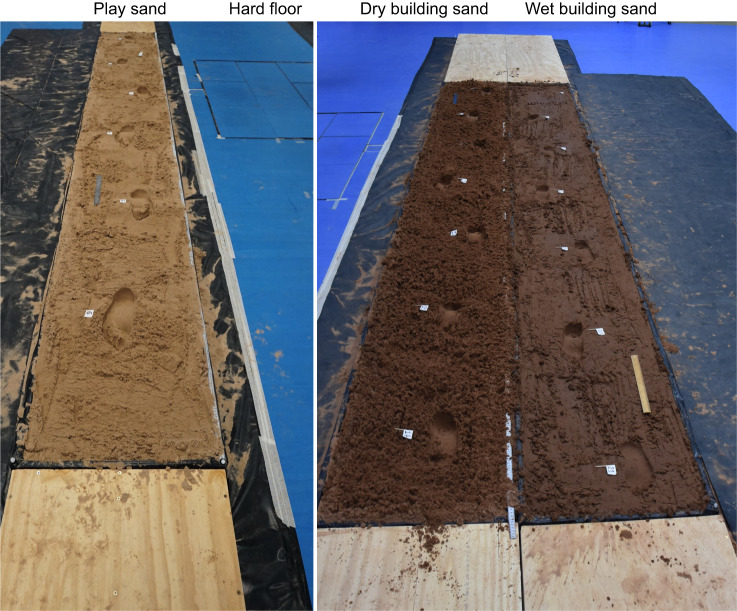
**Example of the setup of the wooden walkways and substrates.** The four different substrates include play sand (far left), hard lab floor (centre left), dry building sand (centre right) and wet building sand (far right).

Twenty-one young, healthy human individuals (9 males, 12 females; age=26.7±5.3 years; height=1.73±0.1 m; body mass=68.5±9.2 kg; body mass index=22.8±2.4 kg m^−2^; see Table 1) signed informed consent before participating in the study in accordance with ethical approval from the University of Liverpool's Central University Research Ethics Committee for Physical Interventions (no. 3757). There were two different types of sand used in this study: finer play sand (Tarmac play pit sand, grain size <1.25 mm) and coarser building sand (Tarmac building sand, grain size <2 mm); both are readily available through commercial suppliers. We also generated two experimental substrates from the building sand by adding different amounts of water, thereby yielding three different substrates in total to compare with locomotion on a hard floor. Our four experimental substrates were therefore: (1) hard floor, (2) wetted building sand, (3) dry building sand and (4) play sand (Fig. 1). To avoid the sand drying out, all sand substrates were wetted, with greater amounts of water added to the wetted building sand. Before initial data collection, the trackways were filled with sand and water was added 1 litre at a time and mixed thoroughly with handheld rakes until the desired wetness was achieved. All three sand walkways were identical in size: 9.6×0.6×0.1 m (length×width×height), including a 4.8 m long middle section filled with sand (Fig. 1). To ensure the sand was comparable as possible for each participant, several measurements were taken prior to all data collection sessions. These involved taking measurements from different points of each walkway using a shear vane (Pilcon hand vane tester) and force gauge (RS Pro RS232), as well as measuring the depth of footprints made by the lead investigator during barefoot walking on each walkway. Before every data collection, the sand was loosened thoroughly using handheld rakes and raked over to create a level surface, then the lead investigator would walk barefoot across each substrate. Excluding the first footprint created, the depths of the footprints were recorded using a ruler at the hallux, midfoot and heel. For the shear vane and force gauge, five values were taken from different points of each walkway (around half-way between each recorded footprint). The shear vane was inserted into the sand to a depth of 50 mm and the force gauge was inserted into the sand to a depth of 30 mm. If the mean values recorded using the different methods were not within the range decided upon *a priori*, the moisture content of the walkways were modified and the steps above would be repeated until these measurements fell within range (Fig. S1), with particular focus on the mean depth of the footprints (Fig. S1D) given the overarching predictions of the study (see above). Values were accepted within ±1 cm for mean footprint depth values, ±8 kN.m^−2^ for mean shear vane measurements and ±0.3 kg for mean force gauge measurements from the objective values.

**Table 1.**
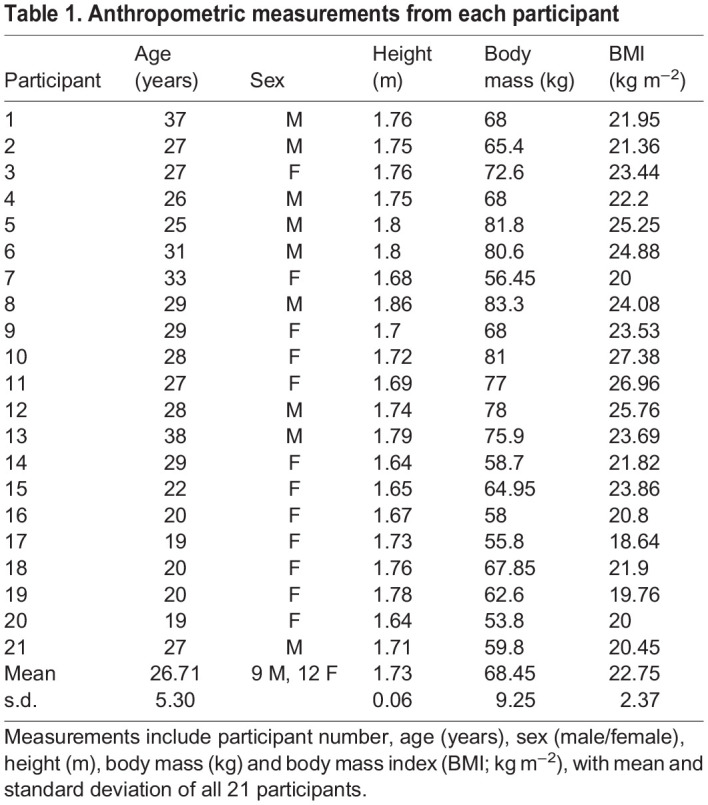
Anthropometric measurements from each participant

On the floor, the participant walked a length of 10 m. Participants performed a total of three trials on the hard lab floor and five trials on each sand walkway with substrate order randomised while 3D kinematics and EMG were measured synchronously. A single trial involved walking at a self-selected speed from one end of the walkway to the other end, always in the same direction. For all trials, whole-body kinematics were recorded at 200 Hz using 69 reflective markers and a 12-camera Qualisys Oqus 7 motion capture system (Qualisys Inc., Götenborg, Sweden). EMGs were recorded using the wireless Trigno EMG (Delsys, MA, USA) system at a sampling rate of 1110 Hz. Standard EMG skin preparation methods (Stegeman and Hermens, 2007) were utilised and electrodes were positioned to record the activity of eight left lower extremity muscles: biceps femoris (BFL), rectus femoris (RF), vastus lateralis (VL), vastus medialis (VM), tibialis anterior (TA), lateral gastrocnemius (LG), medial gastrocnemius (MG) and soleus (SOL). These data collection protocols followed our previous study of human walking on compliant foams (Grant et al., 2022).

### Experimental data processing

Kinematic data processing was undertaken in Visual 3D (C-Motion Inc., Germantown, MD, USA) with a kinematic model comprising 13 segments: feet, shanks, thighs, upper arms and forearms (all of these bilaterally), and head, trunk and pelvis. Kinematic gait events were calculated automatically using a coordinate-based algorithm that used foot positions relative to the pelvis (Zeni et al., 2008), but were also checked manually. From these data, Visual3D calculated CoM motions by incorporating an anthropometric model to calculate segmental CoM positions in relation to the laboratory based on mechanical principle patterns (Hanavan, 1964), which were then exported to MATLAB v.2019a (MathWorks, Natick, MA, USA) to calculate the gravitational potential energy (*E*_pot_), kinetic energy (*E*_kin_) and total mechanical energy (*E*_tot_). Then, the recovery of mechanical energy (expressed as a percentage; *R*), relative amplitude (RA) and congruity (the time when potential energy and kinetic energy are moving in the same direction; CO) were calculated (Cavagna et al., 1976). Hip, knee and ankle joint ranges of motion (ROMs) were calculated from maximum and minimum joint angle values for each individual trial. EMG data processing was undertaken in MATLAB with the raw EMG signals high-pass filtered at 12 Hz with a second-order Butterworth filter, full-wave rectified and cropped to stride. For each muscle, the data were normalised (nEMG) to maximum amplitude during all walking trials for that participant and the integrated values were calculated for each stride (iEMG). These data processing protocols again followed our previous study of human walking on compliant foams (Grant et al., 2022).

### Foot sinking depth

Foot sinking depth was estimated using the *z*-positions of the kinematic markers positioned at the left and right hallux (L/RHALL) and left and right calcaneus (L/RCAL). Before every data collection session, the lab was calibrated with *z*=0 as the height of the lab floor, and markers on each end of the sand walkways were used to calculate the *z*-value of the sand substrates. After data processing, the lowest *z*-values for L/RCAL and L/RHALL for every stride were deducted from the *z*-value of the substrate to estimate the lowest sinking point of the hallux and calcaneus in each substrate. As left and right values were comparable, these were combined for statistical analysis.

### Statistical analyses

Joint ROMs, spatiotemporal data, iEMG data and mechanical energy exchange variables were analysed using linear mixed-effect models (LMMs) (Faraway, 2016), where restricted maximum likelihood was used to assess the significance of the fixed effects, substrate type, sex and speed, in explaining variation with participants set as random effects. All LMMs were performed in R (https://www.r-project.org/) using the lmer function in the R package lme4 (Bates et al., 2015) and lmerTest (Kunzetsova et al., 2017). Joint kinematics were also analysed using one-dimensional statistical parametric mapping (1D-SPM) (Pataky et al., 2013), which allowed for continuous statistical analysis throughout the whole gait cycle. 1D-SPM analyses were performed using MATLAB to compare ankle, knee and hip joint angles across substrates, with a null hypothesis of no difference and an alpha of 0.05. For all spatiotemporal variables, the coefficient of variation (CV; the ratio of the standard deviation to the mean) was calculated as a proxy for gait variability. ANOVA and Tukey's *post hoc* tests performed in R were used to test for significant differences between substrates. Using the R package corrplot (https://CRAN.R-project.org/package=corrplot), Spearman's rank correlations were used to test for relationships between total energy exchange recovery (*R*), iEMG values, spatiotemporal variables and joint ROMs, for each individual trial and ordered according to the first principal component (PC1). Also, Spearman's rank correlations were used to test for relationships between the lowest *z*-positions for calcaneus and hallux and between total energy exchange recovery values (*R*), iEMG values, spatiotemporal variables and joint ROMs, averaged for each individual trial.

## RESULTS

### Foot sinking depths

**Fig. 2.**
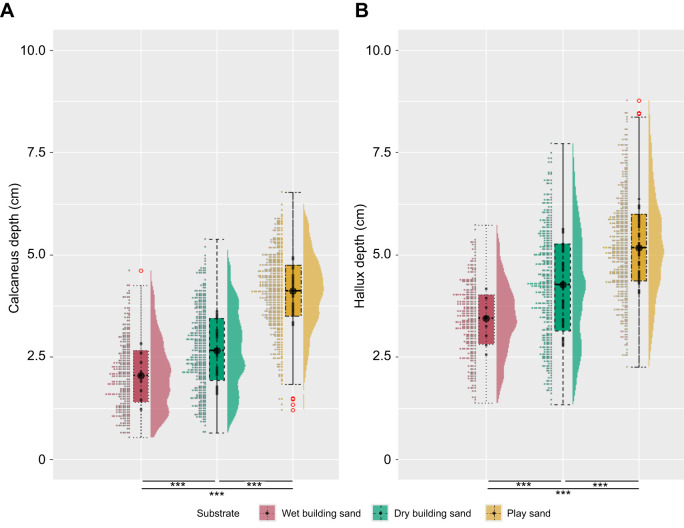
**Distribution of the sinking depth measurements while walking on the three sand substrates.** These values were calculated using the lowest *z*-value positions for every stride for all participants combined (*n*=21) while walking on the sand: wet building sand (red; *n*=295), dry building sand (green; *n*=381) and play sand (yellow; *n*=453). (A) Calcaneus, (B) hallux. Values were used as a proxy for footprint depth. Tukey's *post hoc* tests found significant (*P*<0.001) differences between all substrates for both calcaneus and hallux values.

Fig. 2 shows the foot sinking depths recorded at the left and right calcaneus (L/RCAL) and left and right hallux (L/RHALL) for all participants. The values for L/RCAL (means±s.d.) were 2.08±0.85, 2.68±1.0 and 4.09±0.93 cm on the wet building sand, dry building sand and play sand substrate, respectively (Fig. 2A). The values for L/RHALL were 3.43±0.88, 4.26±1.37 and 5.23±1.24 cm on the wet building sand, dry building sand and play sand substrate, respectively (Fig. 2B). An ANOVA on these values showed that there was a significant (*P*<0.001) difference between substrates. Tukey's *post hoc* analysis showed that there were significant (*P*<0.001) differences between all sand substrates for both calcaneus and hallux values (Table S1A,B).

### Experimental data

**Fig. 3.**
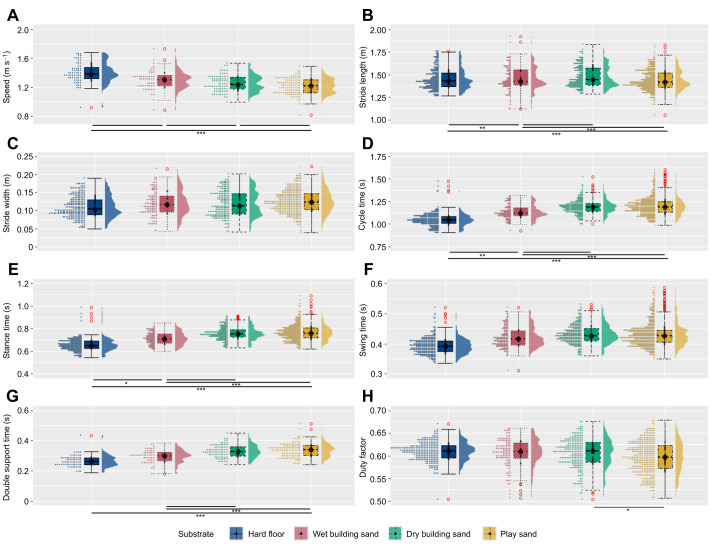
**The distribution of spatiotemporal parameters for all participants combined (*n*=21) while walking on the four different substrates.** Substrates include hard floor (blue; *n*=250), wet building sand (red; *n*=121), dry building sand (green; *n*=215) and play sand (yellow; *n*=347). (A) Speed, (B) stride length, (C) stride width, (D) cycle time, (E) stance time, (F) swing time, (G) double support time and (H) duty factor. Data include all strides for individual trials (*n*=936). Red circles denote an individual stride from any participant that represents a statistical outlier. *P<0.05, **P<0.01, ***P<0.001.

LMMs show that there were significant (*P*<0.05) differences between all substrates for speed, and between most substrates for cycle time, stance time and double limb support time, and some substrates for stride length, swing time and duty factor (Fig. 3; Table S2). There were no significant differences between any substrates for stride width. The CV increased by 12%, 8% and 29% for duty factor and 21%, 11% and 36% for swing time between hard floor and wet building sand, dry building sand and play sand, respectively (Table 2). For speed, stride length, stride width, stance time and double support time, there were both increases and decreases in the CV between different substrates (Table 2). Therefore, there was no clear relationship between the CV and foot sinking depth.

**Table 2.**
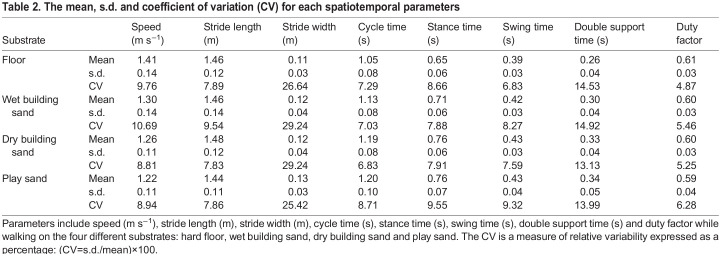
The mean, s.d. and coefficient of variation (CV) for each spatiotemporal parameters

**Fig. 4.**
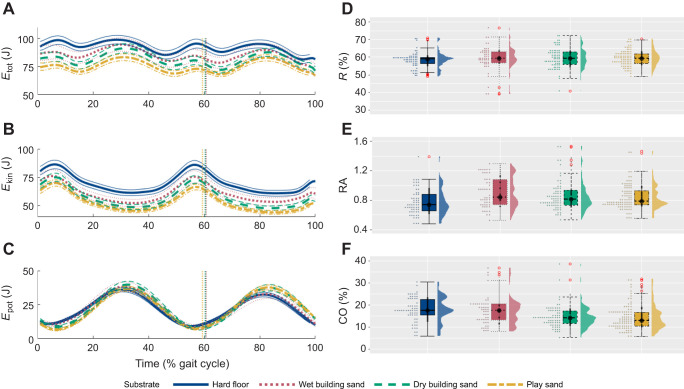
**Mechanical energy exchange variables.** (A) Total mechanical energy (*E*_tot_), (B) kinetic energy (*E*_kin_) and (C) gravitational potential energy (*E*_pot_) of the CoM and normalised to walking stride for participants combined while walking on the four different substrates: hard floor (blue solid), wet building sand (red dotted), dry building sand (green dashed) and play sand (yellow dot-dashed) (mean±95% CI). The distribution of pendulum-like determining variables: (D) the recovery of total energy exchange as a percentage (*R*), (E) relative amplitude (RA) and (F) congruity percentage (CO) for all participants combined (*n*=21) walking on the four different substrates: hard floor (blue; *n*=60), wet building sand (red; *n*=51), dry building sand (green; *n*=74) and play sand (yellow; *n*=88). Red circles denote an individual stride from any participant that represent statistical outlier.

When averaged across participants, *E*_tot_ and *E*_kin_ (Fig. 4A,B) decreased over the whole stride on all sand substrates relative to the hard floor, but particularly on the substrates with greater foot sinkage (dry building sand and play sand; Fig. 2). During most of the stride, *E*_pot_ increased on the sand substrates relative to the hard floor except during toe-off and early stance (Fig. 4C). Relative amplitude (RA) was greater on all sand substrates than on hard floor, but between sands there was a negative correlation between sinking depth and RA, with increases of 15.9%, 10.1% and 8.7% between hard floor and wet building sand, dry building sand and play sand, respectively (Fig. 4E). The recovery of total energy exchange (*R*) increased by 1.7%, 2% and 1.9% between hard floor and wet building sand, dry building sand and play sand, respectively (Fig. 4D). Congruity percentage (CO) decreased by 2.9%, 18.3% and 19.5% between hard floor and wet building sand, dry building sand and play sand, respectively (Fig. 4F). However, LMMs showed that the effect of substrate was not significant (*P*>0.05) for any variables (Table S3).

**Fig. 5.**
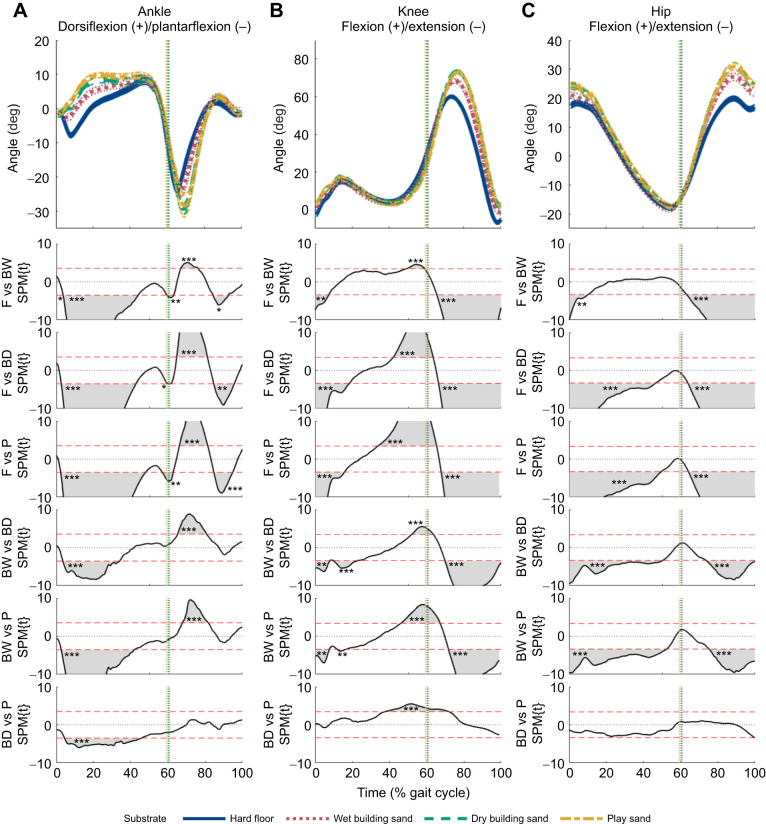
**Ankle, knee and hip joint angles in the sagittal plane for all participants combined (*n*=21) while walking on the four different substrates.** (A) Ankle, (B) knee and (C) hip joints. Substrates include hard floor (blue solid), wet building sand (red dotted), dry building sand (green dashed) and play sand (yellow dot-dashed) (means±95% CI). The vertical dotted lines indicate toe-off. 1D-SPM (utilising paired *t*-tests with Bonferroni corrections) indicates regions of statistically significant differences between walking conditions, when 1D-SPM lines exceed the critical threshold values denoted by the horizontal red dashed lines. Shaded regions (within the SPM graphs) correspond to the period within the gait cycle where walking conditions are statistically significantly different from one another. *P<0.05, **P<0.01, ***P<0.001.

1D-SPM analyses of sagittal plane joint angles found significant differences between most substrates at different stages of the stride (Fig. 5; Table S4). Hip, knee and ankle joint angles were very similar throughout most of the stride on the two sands with the greatest foot sinkage (dry building sand and play sand). During heel-strike, as foot sinkage increased, there was a significant (*P*<0.001) increase in knee and hip flexion (Fig. 5B,C) between all substrates, except between the dry building sand and play sand. During early to mid-stance there was significantly (*P*<0.001) less plantarflexion at the ankle joint (Fig. 5A) between all sand substrates and hard floor, and greater flexion at the hip joint (Fig. 5C) on dry building sand and play sand, compared with both hard floor and wet building sand. During the swing phase, there were significant (*P*<0.001) increases in plantarflexion at the ankle joint and in flexion at the knee and hip joints (Fig. 5A–C) as foot sinkage increased across the substrates. LMMs on joint ROMs (Table S4) found significant (*P*<0.05) substrate effects for ankle joint ROM and some substrate effects for knee joint ROM, but no significant (*P*>0.05) substrate effect for hip joint ROM.

**Fig. 6.**
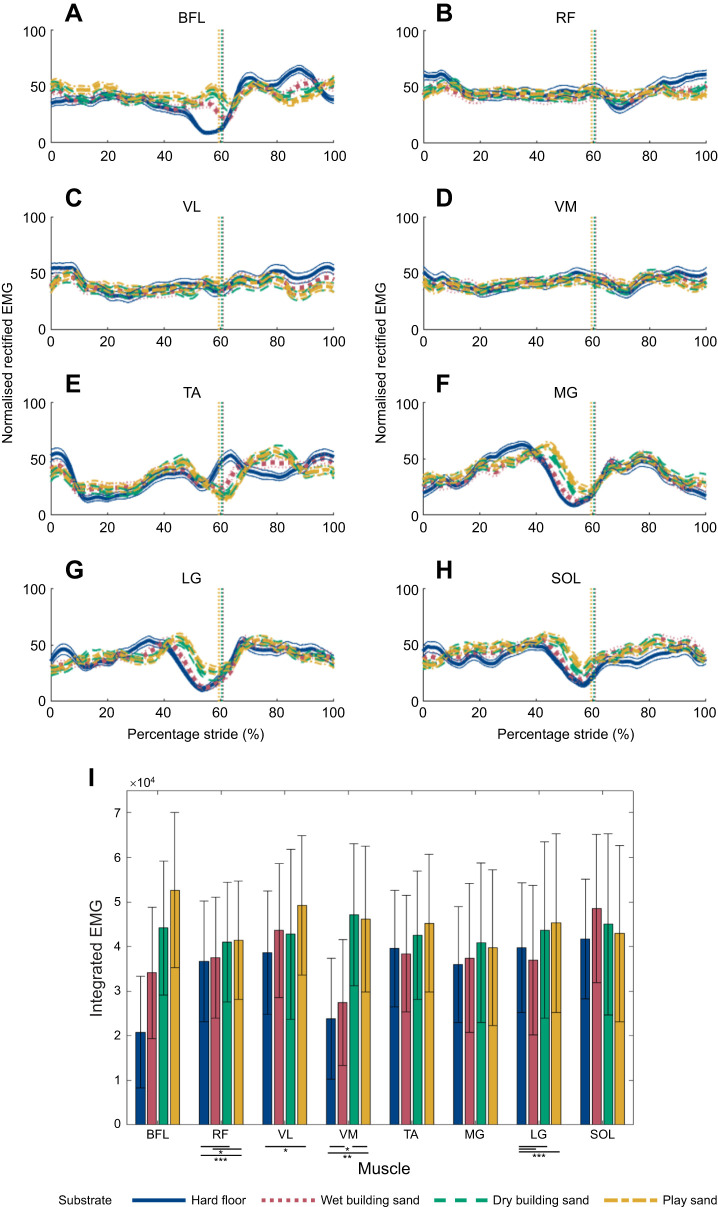
**EMG values for eight left lower extremity muscles for participants combined (*n*=20) while walking on the four different substrates.** Substrates include hard floor (blue), wet building sand (green), dry building sand (red) and play sand (purple). (A–H) normalised rectified EMG amplitudes for: (A) biceps femoris (BFL), (B) rectus femoris (RF), (C) vastus lateralis (VL), (D) vastus medialis (VM), (E) tibialis anterior (TA), (F) lateral gastrocnemius (LG), (G) medial gastrocnemius (MG) and (H) soleus (SOL) (means±95% CI). (I) Integrated EMG (iEMG) values calculated for each stride (mean±s.d.). *P<0.05, **P<0.01, ***P<0.001.

Overall, lower limb muscle activity for all measured muscles were slightly higher as foot sinkage depth increased (Fig. 6). However, there were periods of the stride for all muscles when muscle activations were higher on the hard floor compared with the sand substrates. During heel-strike, nEMG for the RF, VL, VM, TA, LG and SOL were higher on the hard floor than on the sand substrates, but were higher on the sands for the BFL and MG (Fig. 6A–F). During most of the stance phase, nEMG was higher on the sands than the hard floor, except for MG and LG where nEMG was higher on the hard floor during mid-stance. During toe-off, nEMG for the BFL and SOL (Fig. 6A,H) were higher on the sands compared with hard floor, but the TA (Fig. 6E) was higher on the floor. During initial swing, nEMG was higher on the sand substrates than the hard floor for most muscles, except for the BFL and VL (Fig. 6A,C). During mid- to terminal-swing, nEMG was higher on the hard floor for BFL, RF, VL, VM, TA and LG (Fig. 6). iEMG values were higher on sand substrates than the hard floor for all leg muscles (Fig. 6I); however, this did not necessarily relate to an incremental increase in iEMG values as foot sinkage increased across the substrates. LMMs found that there was no significant effect of substrate for the BFL, TA, MG and SOL muscles (Table S5). However, there were significant effects for LG between hard floor and all sand substrates (*P*<0.001) and between hard floor and play sand for VL (*P*<0.05). There were also significant (*P*<0.05) effects between hard floor and both wet building and play sand, and between dry building sand and play sand for VM, and between hard floor and dry building sand and play sand, and between wet building sand and play sand for RF (Fig. 6; Table S5).

LMMs found that speed had a significant (*P*<0.05) effect on all spatiotemporal variables except stride width (Table S2) and total energy exchange (*R*) (Table S3). Sex had a significant (*P*<0.05) effect on some spatiotemporal variables (Table S2) and some iEMG values (Table S5). There were also some significant (*P*<0.05) interaction effects between substrate, sex and speed for most spatiotemporal variables, iEMG values and some joint ROMs.

**Fig. 7.**
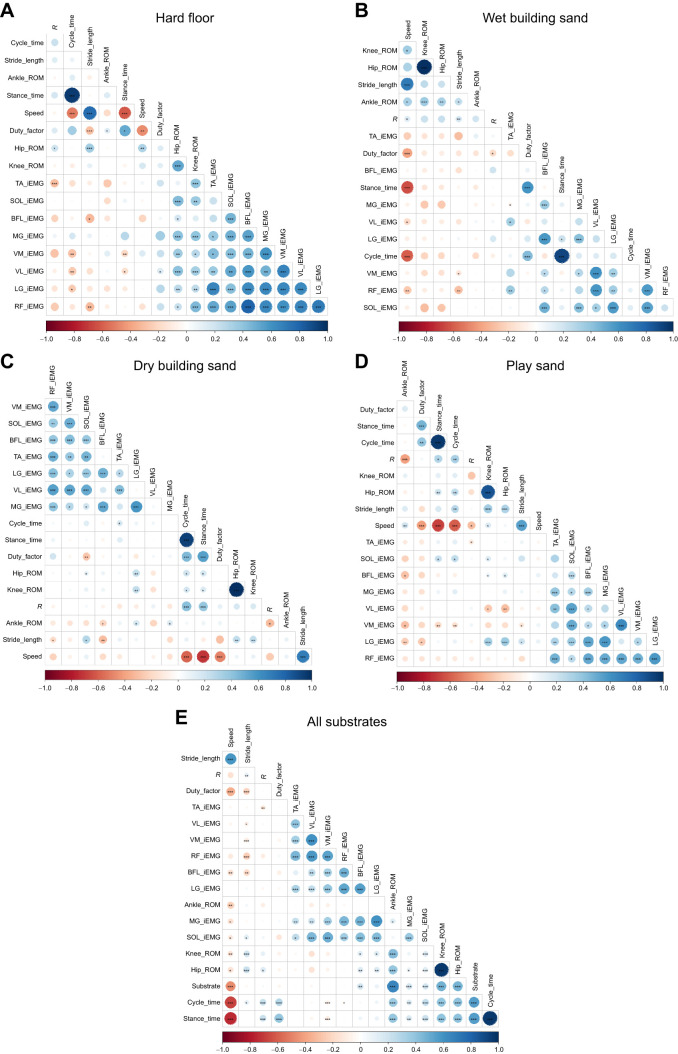
**Correlograms depicting Spearman's rank correlations for pairs of total energy exchange recovery (*R*), spatiotemporal variables, joint ROMs and iEMG variables for all participants combined (*n*=21).** Substrates include (A) hard floor, (B) wet building sand, (C) dry building sand, (D) play sand and (E) all substrates combined. Positive correlations are displayed in blue (max. 1) and negative correlations in red (max. −1) and are ordered by the first principal component. *P<0.05, **P<0.01, ***P<0.001.

The Spearman's rank correlations also showed that there were significant (*P*<0.05) positive correlations between speed and ankle and knee ROM, for wet building sand and play sand, and significant (*P*<0.05) negative correlations between speed and ankle ROM, knee ROM and hip ROM for all substrates combined (Fig. 7). Speed also had significant (*P*<0.001) positive correlations with stride length and significant (*P*<0.01) negative correlations with cycle time, stance time and duty factor for all substrates (Fig. 7). There was also a negative correlation between speed and substrate (Fig. 7E). On all substrates, most iEMG variables had significant (*P*<0.05) positive correlations with other iEMG values but only had a few significant (*P*<0.05) correlations with other variables. There were positive (*P*<0.05) correlations between hip and knee ROM with BFL and LG muscles for play sand (Fig. 7D) and between hip and knee ROM with LG muscles for dry building sand (Fig. 7C). Also, there were negative (*P*<0.05) correlations between hip and knee ROM and VL muscle for play sand (Fig. 7D). There were some significant (*P*<0.05) negative correlations between some muscle activities and spatiotemporal variables such as stance time, cycle time and stride length on different sand substrates, but these were not consistent across the different substrates (Fig. 7B–D). On dry building sand and play sand, there were significant (*P*<0.05) positive correlations between total energy recovery (*R*) and cycle time and stance time. On dry building sand, muscle activities were ordered first by PC1, whereas on the hard floor, wet building sand and play sand, spatiotemporal variables and joint ROM were ordered first (Fig. 7).

**Fig. 8.**
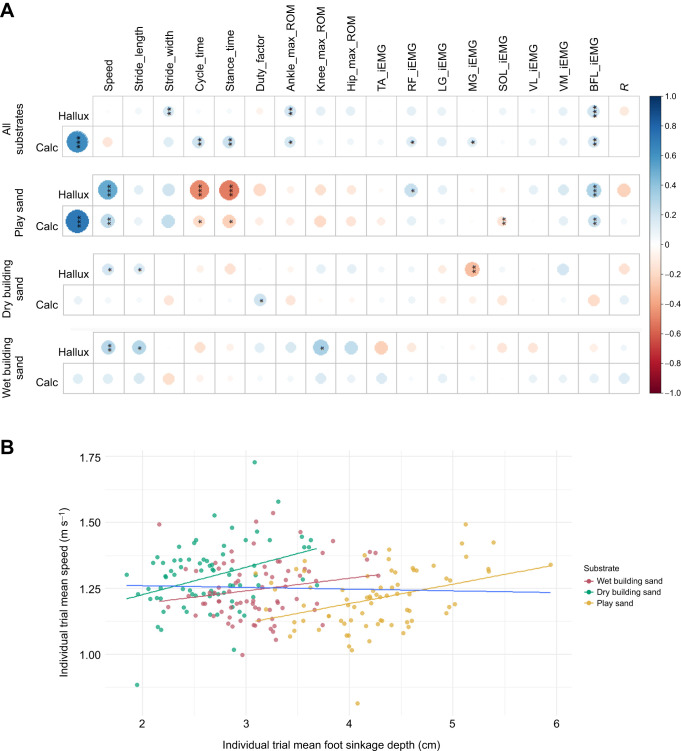
**Correlations between pairs of mean foot sinkage depth values with gait variables for all participants combined (*n*=21).** (A) Correlogram depicting Spearman's rank correlations for pairs of mean calcaneus and hallux values (calculated from lowest *z*-value positions) with spatiotemporal variables, joint ROMs, iEMG variables and total energy exchange recovery (*R*). Positive correlations are displayed in blue (max. 1) and negative correlations in red (max. −1) and are ordered by the first principal component. *P<0.05, **P<0.01, ***P<0.001. (B) Correlations between mean foot sinkage depth (calculated as mean of calcaneus and hallux values) with mean walking speed. Blue line depicts the correlation between mean depth and speed with all substrates combined. Values are means for each trial. Substrates include wet building sand, dry building sand, play sand and all sand substrates combined.

The Spearman's rank correlations recovered relatively few statistically significant relationships between lowest sinking depth (as calculated by *z*-position values at the calcaneus and hallux; Fig. 2) and gait variables (Fig. 8A). In these correlations, a positive relationship indicates that as foot sinking depth increases, the measured variable also increases, whereas negative relationships indicate that as the foot sinking depth increases, the measured variable decreases. There were significant (*P*<0.05) positive correlations between the hallux and speed for all substrates individually (but not with all substrates combined), and significant (*P*<0.05) positive correlations between the calcaneus and speed for play sand (Fig. 8A). However, within each individual sand substrate, foot sinkage depth increased as average walking speed increased over individual trials (Fig. 8B). With all substrates combined, calcaneus depth had significant (*P*<0.05) positive correlations with cycle time, stance time, RF muscle and MG muscle, and hallux had significant (*P*<0.01) positive correlations with stride width and BFL muscle. Both calcaneus and hallux had a significant (*P*<0.05) positive correlation with ankle ROM (Fig. 8A).

## DISCUSSION

Human walking is characterised by centre of mass motion similar to that of an inverted pendulum, with a relatively efficient exchange between kinetic (*E*_kin_) and potential (*E*_pot_) energies of the CoM of the body. P1 stated that pendular energy exchange would have reduced efficiency in substrates with greater overall foot sinkage depth, as proposed by Zamparo et al. (1992). This prediction is not supported by the present data, as there was no significant effect of substrate, with similar values for total energy exchange recovery (*R*), relative amplitude (RA) and congruity (CO) variables (Table S3). We calculated *R* to be 58.4±4.4% on hard floor, 59.4±6.8% on wet building sand, 59.5±5.7% on dry building sand and 59.5±4.4% on play sand (means±s.d.). Similar values were found by Lejeune et al. (1998), with as much as 60% *R* when walking on sand, whereas Zamparo et al. (1992) calculated a relatively lower 43–48% *R* on sand. However, the period of pendular energy exchange typically takes place during mid-stance (Cavagna et al., 2002; Dewolf et al., 2017), possibly after the sand has already been compressed, meaning similar energy exchange variables observed here (Fig. 4) do not necessarily correlate with the amount of mechanical energy exchanged (Bertram and Hasaneini, 2013). Instead, mechanical work has been suggested to be predominately related to the collisional losses that occur during the step-to-step transitions (Donelan et al., 2002; Kuo et al., 2005). Previous studies have suggested that a large portion of energy dissipation occurs immediately after the collision of the human heel with the ground, including the initial impact of heel strike with a second impact as the foot touches down (e.g. Baines et al., 2018; Honert and Zelik, 2019). During these impacts, there is a sudden change in the velocity of the heel/foot as well as the joint (hip, knee and ankle) angular velocities. In this study, we found increased stance times on sand substrates (Fig. 3E); this increase in the collision period would likely result in a reduction in the angular velocity of the foot.

During walking on sand, Jafarnezhadgero et al. (2019) found lower peak posterior ground reaction forces (GRFs) during heel contact and lower peak anterior GRFs during push-off, compared with stable ground. Owing to sand deformation, the foot is prevented from plantarflexion during early stance as observed by increased ankle dorsiflexion (Fig. 5A), which likely resulted in greater energy dissipation. Overall, it may be inferred that during walking on sand, collisional losses may be comparatively lower relative to hard floor, but there will be an increase in energy loss from substrate deformation, requiring greater mechanical work to offset this loss. However, this may not necessarily be through increased muscle activation, as suggested by the similar muscle activations found on all substrates (Fig. 6). Studies on walking on compliant foam mats (MacLellan and Patla, 2006) and on sand (Svenningsen et al., 2019) found that vertical CoM decreased to provide a more stable posture, and during walking on uneven surfaces, Voloshina et al. (2013) found participants adopted a more crouched gait, which lowered the body CoM at the expense of increased mechanical work. However, there is no evidence that participants in this study adopted a similar strategy, as there were little differences in knee and hip flexion (Fig. 5B,C) between all substrates during stance.

Locomotion on complex, uneven or compliant substrates can affect stability and requires the human body to adapt by changing gait mechanisms (Darici and Kuo, 2023; Gates et al., 2012; Hak et al., 2012; MacLellan and Patla, 2006; Voloshina et al., 2013). The second prediction (P2) stated that stance time and stride width would increase and walking speed and stride length would decrease on substrates with relatively greater average foot sinkage depths. This prediction is partially supported by the present data (Fig. 3; Table S2). Participants adopted a significantly slower walking speed on substrates with greater foot sinkage depths (Fig. 3; Table S2). This may represent an attempt to increase stability on the more deformable surface, a need for more accurate foot placement (Matthis et al., 2018), or be a product of greater deceleration during ground contact on sand (Bates et al., 2013). As walking speed influences many variables measured in this study (Figs 7, 8; Tables S3–S5), the reduction in walking speed on sands with greater average sinkage depths may be not only caused by greater instability due to the deformable nature of the surface, but also a necessary adjustment to reduce increased mechanical costs associated with these various gait adaptations (although see discussions of depth correlations below). Results here showed significant increases in cycle time, stance time and double limb support time between the two sand substrates that yielded the greatest foot sinkage (dry building sand and play sand) and the other two substrates (hard floor and wet building sand) (Fig. 3; Table S2). Yet, duty factor was similar for all substrates, suggesting relative stance and swing times were similar. The observed increase in cycle time but similar duty factor on all substrates may be related to energy storage and release mechanisms, particularly on substrates with greater overall foot sinking depth (Figs 7, 8). Our results show that there was no significant difference in stride width between any substrates (Table S2), but there were significant differences in stride length (Fig. 3). Wider and longer steps would require more mechanical work, and therefore increase metabolic costs, to redirect the CoM between steps (Donelan et al., 2002). The participants' step width and length may be based on a trade-off between minimising mechanical work and the cost of active stabilisation of lateral balance during locomotion on the sands.

For walking on irregular and compliant substrates, previous studies have shown that participants display greater hip and knee flexion during the swing phase, resulting in greater mechanical work (Gates et al., 2012; Grant et al., 2022; Marigold and Patla, 2002; Pinnington et al., 2005; Svenningsen et al., 2019; Voloshina et al., 2013). Furthermore, during the stance phase of walking on sand, the foot sinks and often slips backwards as the sand is displaced. This is observed during jumping on sand, where slipping caused an increased range of motion at the ankle joint prior to push-off (Giatsis et al., 2004). Our third prediction (P3) stated that on substrates with greater sinkage depths there would be greater joint excursions at the hip, knee and ankle joints. This prediction is supported by the present data (Fig. 5; Tables S3, S4). Our results show that hip and knee flexion (Fig. 5B,C) were significantly greater on substrates with greater average foot sinkage depths, in agreement with previous studies on locomotion on other types of compliant substrates (Grant et al., 2022; Pinnington et al., 2005; Svenningsen et al., 2019). On the sands, there were also greater ROMs at the ankle joint throughout the stride (Fig. 5A) and significant (*P*<0.05) positive correlations between ankle ROM and substrate (Fig. 7E). The greater ankle dorsiflexion at early stance is most likely due to the sinking of the heel into the substrate after heel-strike as there is no significant difference in ankle joint angle between any substrates in late stance. Reduced ankle plantarflexion has been associated with greater positive work by the joints and increased overall metabolic energy expenditure (Huang et al., 2015). Therefore, the greater ankle dorsiflexion observed on sand may result in reduced energy storage potential in the ankle plantar flexors during stance, limiting the amount of energy available for recovery and propulsion during push-off, resulting in increased mechanical work. During the swing phase, greater hip and knee flexion and greater ankle plantarflexion are likely to ensure toe clearance on the compliant sand substrates, as seen during locomotion on irregular surfaces (Merryweather et al., 2011; Svenningsen et al., 2019) and compliant foam (Grant et al., 2022). The increase in hip and knee flexion in the trailing leg during swing (Fig. 5) may also represent a compensatory action for the loss of momentum at the stance leg owing to sand deformation, and allow a greater horizontal GRF to be exerted against the sand substrate to negate potential energy lost owing to foot slippage during push-off (Lejeune et al., 1998; Zamparo et al., 1992).

Previous studies have suggested that walking on uneven, irregular or compliant terrain incurs increased mechanical work at the knee and hip owing to greater knee and hip flexion, particularly during the swing phase (Gates et al., 2012; Grant et al., 2022; Voloshina et al., 2013). Furthermore, when walking on sand, the muscles in the leg may need to produce additional work in order to ensure stability owing to surface displacement underfoot (Lejeune et al., 1998; Zamparo et al., 1992). The fourth prediction (P4) stated that there would be greater muscle activation of lower limb muscles on substrates with greater average sinking depth. This prediction is generally not supported by the present data. Overall, all lower limb muscle activities (nEMG) increased slightly on substrates with greater overall foot sinking depth (Fig. 6), but these differences were often not statistically significant (Fig. 6; Table S5). However, there was a significant effect of substrate for the RF, VL, VM and LG muscles, mostly between hard floor and dry building sand/play sand (Fig. 6; Table S5), which may be associated with the increased ROMs at the joints (Fig. 5). Pinnington et al. (2005) reported similar kinematics to this study but found increased activations during running on sand, with EMG activation of the hamstrings and quadriceps nearly two times higher on sand than on a firm surface. During locomotion on stable ground, changes in speed have been shown to be accompanied by numerous changes to gait variables such as increased step length and cycle time and decreased stance time (Nilsson et al., 1985), and changes to muscle co-activation patterns (Fiori et al., 2024). Furthermore, it has been shown that average positive mechanical work increases with running speed (Cavagna and Kaneko, 1977) and walking speed (Farris and Sawicki, 2012). Therefore, changes in muscle activation when walking and running on sand versus hard substrates may not be parallel.

Lejeune et al. (1998) found that when walking on sand, more work was done on the substrate by the foot owing to foot slippage during push-off. Peak ankle power results from a combination of elastic recoil of the Achilles tendon and active muscle contraction of the triceps surae muscles. Postural disturbances owing to slipping will result in muscles actively contracting to ensure stabilisation, particularly in the gastrocnemius and soleus muscles responsible for ankle plantarflexion (Farris et al., 2020; Kelly et al., 2015). Bates et al. (2013) suggested that walking on compliant substrates requires greater muscle–tendon forces from the ankle extensors to generate the propulsion needed from mid-stance to reaccelerate into the swing phase. Although there were slightly higher activations of the MG (Fig. 6F) and the SOL (Fig. 6H) on the sands during the propulsive phase of stride compared with the floor, these differences were not found to be statistically significant (Table S5). However, there were significant increases in LG (Fig. 6G) between the hard floor and dry building sand and play sand. These changes in iEMG values and greater flexion at the joints could potentially relate to greater metabolic costs of locomotion often observed on compliant or deformable substrates, but would not necessarily be associated with changes in mechanical costs of locomotion (Bertram and Hasaneini, 2013; Mian et al., 2006). Also, the increase in stance time during walking on sand (Fig. 3E) may suggest participants could alter their motor recruitment patterns with an increase in prolonged activation throughout stance. Furthermore, some joint work may be performed passively through elastic energy storage and return by the tendons and foot muscles, which were not measured in this study. For example, greater ankle dorsiflexion observed during stance on the sands (Fig. 5A) could increase tension in the Achilles tendon (Mann and Hagy, 1980).

Our fifth and sixth predictions sought to test for correlations between changes in gait metrics within and across all four substrates (P5), and between gait metrics and foot sinkage depth within and across the three sand substrates (P6). Support for these predictions would suggest mechanistic relationships between gait variables, and that foot sinkage depth has a strong causative effect on those relationships. Overall, we found support for P5, with a multitude of variables showing statistically significant correlations within and across the hard floor and three sand substrates (Fig. 7). On the dry building sand, correlations between the activity of individual muscles showed the strongest correlations, whereas on other substrates, correlations between spatiotemporal and joint kinematics variables were strongest (Fig. 7). In contrast, we found weak support for P6, with relatively few statistically significant correlations between foot sinkage depth and gait metrics either within individual sand substrates or across them as a whole (Fig. 8A). One possible reason for the lack of more widespread significant relationships between gait metrics and foot sinkage depth is our use of average data per trial as inputs into correlation tests. This averaging of data may be sufficient to detect correlations between individual gait parameters themselves (P5), particularly when the hard floor is included (Fig. 7E), but may remove important within-trial or step-to-step relationships between gait metrics and foot sinkage on the sands. However, analysing these interactions on a step-by-step basis is not necessarily straightforward, as limb–substrate mechanics in one step are likely to be influenced by the preceding (temporally overlapping) step and thus attributing a single footprint depth to gait metric values from a single, discrete period of time in a simple correlation test may also be limiting.

Variations in walking speed are also clearly an important component of variation in gait metrics and foot sinking depths recovered here. Speed is recovered as an important fixed effect in some LMMs and it was found to significantly correlate with sinkage depth within individual sand substrates, but not across them overall (Fig. 8A). This latter finding reflects the fact although sinkage depth showed a slight negative correlation with speed across substrates (i.e. the slowest mean walking speeds yield the deepest sinkage depths on average; Fig. 8A), we found that within each individual sand substrate, foot sinkage depths showed positive relationships with speed (i.e. trials with higher mean walking speeds yielded greater foot sinkage on average within each substrate; Fig. 8B). One logical interpretation of this within-substrate trend is that faster walking speeds and their associated changes with spatiotemporal variables (e.g. shorter stance times) result in higher GRFs and greater displacement of sand upon contact. However, this potential mechanistic relationship appears detached from the majority of joint kinematic and EMG metrics analysed here. For example, LMM models only rarely recovered significant effects of speed on joint kinematic variables, and significant relationships with speed (Fig. 7) and sinkage depth (Fig. 8) were also rarely found in correlation tests (Fig. 8A). Indeed, here we found significant increases in joint excursions (Fig. 5) on substrates where walking speed was on average reduced (Fig. 3), which directly juxtaposes the widely recovered tendency for joint excursions to increase with increasing speed on hard substrates (Fukuchi et al., 2019; Kirtley et al., 1985; Oberg et al., 1993). This suggests that the potential causative relationship between foot sinkage depth and walking speed within sand substrates is not coupled with systematic changes in joint kinematics and muscle activity in the same way as is observed across sand substrates (Figs 5–7).

High levels of inter-participant variation also represent a complicating factor in our analyses. Previous studies have found that participant variability increased when walking over more complex, uneven or compliant substrates (Donelan et al., 2002; MacLellan and Patla, 2006; Marigold and Patla, 2002). In this study, with a relatively homogeneous population, we observed considerable inter- and intra-participant variability for most variables measured in this study. LMMs found that a high proportion of the variance shown in the measured variables were due to the random effects (participants) rather than the fixed effects (substrate, speed and sex) (Table S2–S6), and many variables displayed large ranges and standard deviations. However, the CVs for most spatiotemporal variables were similar across substrates (Table 2). Some variables increased whereas others decreased in CV on sand compared with the hard floor, but these differences did not appear to correlate with increased foot sinkage as there were large but non-systematic differences in CV for some spatiotemporal variables between the two sand substrates that yielded the greatest foot sinkage, dry building sand and play sand (Table 2). Although influences of participant, sex and speed have been included in the statistical models, investigating sex differences and individual participant differences when walking over different compliant substrates may be an interesting and useful area of research in the future.

Overall, our findings suggest that when walking over natural compliant substrates such as sand that differ in overall foot sinking depth, humans will modify their gait strategies in response to a potential increase in energy lost to the deformable substrate, and subsequent increase in mechanical work. These results not only enhance our understanding of how walking in humans is modulated on deformable natural terrains, but also have implications for the study of the evolution of human bipedalism from fossil footprints. Our quantitative demonstration that humans alter numerous joint and spatiotemporal kinematic variables due to overall foot sinking depth suggests that comparisons of locomotor evolution are most appropriate where footprint depth across footprint sites were similar. Here, we generally recovered greater differences between wet and dry building sand than between the dry building sand and dry play sand types, where average foot sinkage was deeper. Interestingly, with increasing foot sinkage we found that participants employed greater overall flexed hip and knee postures, as well as greater ankle dorsiflexion. The evolution of bipedalism in the human lineage is marked by a shift from more flexed joint ROMs (particularly at the hip and knee, so-called bent-hip bent-knee gait) as seen in extant non-human apes to more extended or upright postures seen in modern humans (Crompton et al., 2023; Harcourt-Smith, 2010; Pontzer et al., 2014). Assuming extinct bipedal hominids responded qualitatively similarly to modern humans when faced with compliant substrates, our results suggest that temporal comparisons of footprint sites with highly disparate depth profiles could lead to erroneous interpretations about relative limb postures of track makers and the appearance of a fully upright modern human gait.

### Conclusions

The results of this study provide new insights into the understanding of human walking and substrate–foot interactions on deformable substrates. On the sands, participants displayed greater ROMs at the hip, knee and ankle joints, primarily owing to greater peak flexion at the hip and knee joints during swing and greater ankle dorsiflexion during stance, compared with the hard floor (Fig. 5). Furthermore, participants adopted a slower walking speed and increased cycle time, stance time and swing time on all sand substrates relative to the hard floor (Fig. 3). Most gait changes were similar on the two substrates with greater overall sinking depth – dry building sand and play sand – with the wet building sand sand as an intermediate between these and the hard floor. In contrast, we found no evidence for differences in pendular energy recovery across substrates (Fig. 4), and only modest changes in muscle activations were observed (Fig. 6). We found frequent correlated changes between these metrics both within and between the four studied substrates, suggesting a degree of coupled or mechanistic interactions in their variation (Fig. 7). However, although substrates that on average show different foot sinkage depths yield the aforementioned differences in spatiotemporal and joint kinematics (Figs 3, 5, 6), we recovered relatively few significant correlations between foot sinkage depth and gait metrics at the level of individual trials, either within or across substrates (Fig. 8A). Within substrates, we recovered evidence of a causative relationship between foot sinkage depth and speed, which appears independent of joint kinematics and muscle activity (Fig. 8). However, future work is required to confirm whether this potential relationship is observed during locomotion on other deformable substrates.

## Supplementary Material

10.1242/jexbio.246787_sup1 Supplementary information

## References

[JEB246787C48] Baines, P. M., Schwab, A. L. and van Soest, A. J. (2018). Experimental estimation of energy absorption during heel strike in human barefoot walking. *PloS One* 13, e0197428. 10.1371/journal.pone.0197428 29953479 PMC6023236

[JEB246787C1] Bates, K. T., Savage, R., Pataky, T. C., Morse, S. A., Webster, E., Falkingham, P. L., Ren, L., Qian, Z., Collins, D., Bennett, M. R. et al. (2013). Does footprint depth correlate with foot motion and pressure? *J. R. Soc. Interface* 10, 20130009. 10.1098/rsif.2013.0009 23516064 PMC3645411

[JEB246787C2] Bates, D., Mächler, M., Bolker, B. and Walker, S. (2015). Fitting linear mixed-effects models using lme4. *J. Stat. Softw.* 67, 1-48. 10.18637/jss.v067.i01

[JEB246787C3] Bertram, J. E. A. and Hasaneini, S. J. (2013). Neglected losses and key costs: tracking the energetics of walking and running. *J. Exp. Biol.* 216, 933-938. 10.1242/jeb.078543 23447662

[JEB246787C4] Cavagna, G. A. and Kaneko, M. (1977). Mechanical work and efficiency in level walking and running. *J. Physiol.* 268, 467-481. 10.1113/jphysiol.1977.sp011866 874922 PMC1283673

[JEB246787C5] Cavagna, G. A., Thys, H. and Zamboni, A. (1976). The sources of external work in level walking and running. *J. Physiol.* 262, 639-657. 10.1113/jphysiol.1976.sp011613 1011078 PMC1307665

[JEB246787C49] Cavagna, G. A., Willems, P. A., Legramandi, M. A. and Heglund, N. C. (2002). Pendular energy transduction within the step in human walking. *J. Exp. Biol.* 205, 3413-3422. 10.1242/jeb.205.21.3413 12324550

[JEB246787C6] Crompton, R. H., Sellers, W., Davids, K. and McClymont, J. (2023). Biomechanics and the origins of human bipedal walking: the last 50 years. *J. Biomech.* 157, 111701. 10.1016/j.jbiomech.2023.111701 37451208

[JEB246787C7] Darici, O. and Kuo, A. D. (2023). Humans plan for the near future to walk economically on uneven terrain. *Proc. Natl. Acad. Sci. USA* 120, e2211405120. 10.1073/pnas.2211405120 37126717 PMC10175744

[JEB246787C8] Davies, S. E. H. and Mackinnon, S. N. (2006). The energetics of walking on sand and grass at various speeds. *Ergonomics* 49, 651-660. 10.1080/00140130600558023 16720526

[JEB246787C50] Dewolf, A. H., Ivanenko, Y. P., Lacquaniti, F. and Willems, P. A. (2017). Pendular energy transduction within the step during human walking on slopes at different speeds. *PloS One* 12, e0186963. 10.1371/journal.pone.0186963 29073208 PMC5658120

[JEB246787C9] Donelan, J. M., Kram, R. and Kuo, A. D. (2002). Mechanical work for step-to-step transitions is a major determinant of the metabolic cost of human walking. *J. Exp. Biol.* 205, 3717-3727. 10.1242/jeb.205.23.3717 12409498

[JEB246787C10] Faraway, J. J. (2016). *Extending the Linear Model with R: Generalized Linear, Mixed Effects and Nonparametric Regression Models*. Chapman and Hall/CRC.

[JEB246787C11] Farris, D. J. and Sawicki, G. S. (2012). The mechanics and energetics of human walking and running: a joint level perspective. *J. R. Soc. Interface* 9, 110-118. 10.1098/rsif.2011.0182 21613286 PMC3223624

[JEB246787C12] Farris, D. J., Birch, J. and Kelly, L. A. (2020). Foot stiffening during the push-off phase of human walking is linked to active muscle contraction, and not the windlass mechanism. *J. R. Soc. Interface* 17, 20200208. 10.1098/rsif.2020.0208 32674708 PMC7423437

[JEB246787C13] Fiori, L., Castiglia, S. F., Chini, G., Draicchio, F., Sacco, F., Serrao, M., Tatarelli, A., Varrecchia, T. and Ranavolo, A. (2024). The lower limb muscle co-activation map during human locomotion: from slow walking to running. *Bioengineering* 11, 288. 10.3390/bioengineering11030288 38534562 PMC10968304

[JEB246787C14] Fukuchi, C. A., Fukuchi, R. K. and Duarte, M. (2019). Effects of walking speed on gait biomechanics in healthy participants: a systematic review and meta-analysis. *Syst. Rev.* 8, 153. 10.1186/s13643-019-1063-z 31248456 PMC6595586

[JEB246787C15] Gates, D. H., Wilken, J. M., Scott, S. J., Sinitski, E. H. and Dingwell, J. B. (2012). Kinematic strategies for walking across a destabilizing rock surface. *Gait Posture* 35, 36-42. 10.1016/j.gaitpost.2011.08.001 21890361 PMC3262902

[JEB246787C16] Giatsis, G., Kollias, I., Panoutsakopoulos, V. and Papaiakovou, G. (2004). Biomechanical differences in elite beach-volleyball players in vertical squat jump on rigid and sand surface. *Sports Biomech.* 3, 145-158. 10.1080/14763140408522835 15079993

[JEB246787C17] Grant, B., Charles, J., Geraghty, B., Gardiner, J., D'Août, K., Falkingham, P. L. and Bates, K. T. (2022). Why does the metabolic cost of walking increase on compliant substrates? *J. R. Soc. Interface* 19, 20220483. 10.1098/rsif.2022.0483 36448287 PMC9709563

[JEB246787C18] Hak, L., Houdijk, H., Steenbrink, F., Mert, A., Van Der Wurff, P., Beek, P. J. and Van Dieën, J. H. (2012). Speeding up or slowing down? Gait adaptations to preserve gait stability in response to balance perturbations. *Gait Posture* 36, 260-264. 10.1016/j.gaitpost.2012.03.005 22464635

[JEB246787C19] Hanavan, E. P. Jr (1964). *A Mathematical Model of the Human Body*. Air Force Aerospace Medical Research Lab, Wright-Patterson AFB, OH, USA. 14243640

[JEB246787C20] Harcourt-Smith, W. H. E. (2010). The first hominins and the origins of bipedalism. *Evol. Educ. Outreach* 3, 333-340. 10.1007/s12052-010-0257-6

[JEB246787C21] Holowka, N. B., Kraft, T. S., Wallace, I. J., Gurven, M. and Venkataraman, V. V. (2022). Forest terrains influence walking kinematics among indigenous Tsimane of the Bolivian Amazon. *Evol. Hum. Sci.* 4, e19. 10.1017/ehs.2022.13 37588935 PMC10426037

[JEB246787C53] Honert, E. C. and Zelik, K. E. (2019). Foot and shoe responsible for majority of soft tissue work in early stance of walking. *Human Movement Sci.* 64, 191-202. 10.1016/j.humov.2019.01.008 PMC8777387 30769210

[JEB246787C22] Huang, T. W., Shorter, K. A., Adamczyk, P. G. and Kuo, A. D. (2015). Mechanical and energetic consequences of reduced ankle plantar-flexion in human walking. *J. Exp. Biol.* 218, 3541-3550. 10.1242/jeb.113910 26385330 PMC4664043

[JEB246787C52] Jafarnezhadgero, A., Fatollahi, A., Amirzadeh, N., Siahkouhian, M. and Granacher, U. (2019). Ground reaction forces and muscle activity while walking on sand versus stable ground in individuals with pronated feet compared with healthy controls. *PloS One* 14, e0223219. 10.1371/journal.pone.0223219 31557258 PMC6762175

[JEB246787C23] Kelly, L. A., Lichtwark, G. A. and Cresswell, A. G. (2015). Active regulation of longitudinal arch compression and recoil during walking and running. *J. R Soc. Interface* 12, 20141076. 10.1098/rsif.2014.1076 25551151 PMC4277100

[JEB246787C24] Kerdok, A. E., Biewener, A. A., McMahon, T. A., Weyand, P. G. and Herr, H. M. (2002). Energetics and mechanics of human running on surfaces of different stiffnesses. *J. Appl. Physiol.* 92, 469-478. 10.1152/japplphysiol.01164.2000 11796653

[JEB246787C25] Kirtley, C., Whittle, M. W. and Jefferson, R. J. (1985). Influence of walking speed on gait parameters. *J. Biomed. Eng.* 7, 282-288. 10.1016/0141-5425(85)90055-X 4057987

[JEB246787C26] Kunzetsova, A., Brockhoff, P. and Christensen, R. (2017). lmerTest package: tests in linear mixed effect models. *J. Stat. Softw.* 82, 1-26. 10.18637/jss.v082.i13

[JEB246787C27] Kuo, A. D., Donelan, J. M. and Ruina, A. (2005). Energetic consequences of walking like an inverted pendulum: step-to-step transitions. *Exerc. Sport Sci. Rev.* 33, 88-97. 10.1097/00003677-200504000-00006 15821430

[JEB246787C28] Lejeune, T. M., Willems, P. A. and Heglund, N. C. (1998). Mechanics and energetics of human locomotion on sand. *J. Exp. Biol.* 201, 2071-2080. 10.1242/jeb.201.13.2071 9622579

[JEB246787C29] MacLellan, M. J. and Patla, A. E. (2006). Adaptations of walking pattern on a compliant surface to regulate dynamic stability. *Exp. Brain Res.* 173, 521-530. 10.1007/s00221-006-0399-5 16491406

[JEB246787C51] Mann, R. A. and Hagy, J. (1980). Biomechanics of walking, running, and sprinting. *Am. J. Sports Med.* 8, 345-350. 10.1177/036354658000800510 7416353

[JEB246787C30] Marigold, D. S. and Patla, A. E. (2002). Strategies for dynamic stability during locomotion on a slippery surface: effects of prior experience and knowledge. *J. Neurophysiol.* 88, 339-353. 10.1152/jn.00691.2001 12091559

[JEB246787C31] Matthis, J. S., Yates, J. L. and Hayhoe, M. M. (2018). Gaze and the control of foot placement when walking in natural terrain. *Curr. Biol.* 28, 1224-1233.e5. 10.1016/j.cub.2018.03.008 29657116 PMC5937949

[JEB246787C32] Merryweather, A., Yoo, B. and Bloswick, D. (2011). Gait characteristics associated with trip-induced falls on level and sloped irregular surfaces. *Minerals* 1, 109-121. 10.3390/min1010109

[JEB246787C33] Mian, O. S., Thom, J. M., Ardigò, L. P., Narici, M. V. and Minetti, A. E. (2006). Metabolic cost, mechanical work, and efficiency during walking in young and older men. *Acta Physiol.* 186, 127-139. 10.1111/j.1748-1716.2006.01522.x 16497190

[JEB246787C34] Nilsson, J., Thorstensson, A. and Halbertsma, J. (1985). Changes in leg movements and muscle activity with speed of locomotion and mode of progression in humans. *Acta Physiol. Scand.* 123, 457-475. 10.1111/j.1748-1716.1985.tb07612.x 3993402

[JEB246787C35] Oberg, T., Karsznia, A. and Oberg, K. (1993). Basic gait parameters: reference data for normal subjects, 10–79 years of age. *J. Rehabil. Res. Dev.* 30, 210-223. 8035350

[JEB246787C36] Pandolf, K. B., Haisman, M. F. and Goldman, R. F. (1976). Metabolic energy expenditure and terrain coefficients for walking on snow. *Ergonomics* 19, 683-690. 10.1080/00140137608931583 1009916

[JEB246787C37] Pataky, T. C., Robinson, M. A. and Vanrenterghem, J. (2013). Vector field statistical analysis of kinematic and force trajectories. *J. Biomech.* 46, 2394-2401. 10.1016/j.jbiomech.2013.07.031 23948374

[JEB246787C38] Peyré-Tartaruga, L. A. and Coertjens, M. (2018). Locomotion as a powerful model to study integrative physiology: efficiency, economy, and power relationship. *Front. Physiol.* 9, 1789. 10.3389/fphys.2018.01789 30618802 PMC6297284

[JEB246787C39] Pinnington, H. C. and Dawson, B. (2001). The energy cost of running on grass compared to soft dry beach sand. *J. Sci. Med. Sport* 4, 416-430. 10.1016/S1440-2440(01)80051-7 11905936

[JEB246787C40] Pinnington, H. C., Lloyd, D. G., Besier, T. F. and Dawson, B. (2005). Kinematic and electromyography analysis of submaximal differences running on a firm surface compared with soft, dry sand. *Eur. J. Appl. Physiol.* 94, 242-253. 10.1007/s00421-005-1323-6 15815938

[JEB246787C41] Pontzer, H., Raichlen, D. A. and Rodman, P. S. (2014). Bipedal and quadrupedal locomotion in chimpanzees. *J. Hum. Evol.* 66, 64-82. 10.1016/j.jhevol.2013.10.002 24315239

[JEB246787C42] Soule, R. G. and Goldman, R. F. (1972). Terrain coefficients for energy cost prediction. *J. Appl. Physiol.* 32, 706-708. 10.1152/jappl.1972.32.5.706 5038861

[JEB246787C43] Stegeman, D. and Hermens, H. (2007). Standards for surface electromyography: the European project Surface EMG for non-invasive assessment of muscles (SENIAM). *Enschede: Roessingh Res. Dev.* 10, 8-12.

[JEB246787C44] Svenningsen, F. P., De Zee, M. and Oliveira, A. S. (2019). The effect of shoe and floor characteristics on walking kinematics. *Hum. Mov. Sci.* 66, 63-72. 10.1016/j.humov.2019.03.014 30921761

[JEB246787C45] Voloshina, A. S., Kuo, A. D., Daley, M. A. and Ferris, D. P. (2013). Biomechanics and energetics of walking on uneven terrain. *J. Exp. Biol.* 216, 3963-3970. 10.1242/jeb.081711 23913951 PMC4236228

[JEB246787C46] Zamparo, P., Perini, R., Orizio, C., Sacher, M. and Ferretti, G. (1992). The energy cost of walking or running on sand. *Eur. J. Appl. Physiol. Occup. Physiol.* 65, 183-187. 10.1007/BF00705078 1327762

[JEB246787C47] Zeni, J. Jr, Richards, J. and Higginson, J. (2008). Two simple methods for determining gait events during treadmill and overground walking using kinematic data. *Gait Posture* 27, 710-714. 10.1016/j.gaitpost.2007.07.007 17723303 PMC2384115

