# Supplementary material for: Human walking biomechanics on sand substrates of varying foot sinking depth
Source: J Exp Biol. 2024 Nov 5;227(21):jeb246787. doi: 10.1242/jeb.246787 (PMC11574363; doi:10.1242/jeb.246787)
Supplement: Supplementary information [file jexbio-227-246787-s1.pdf]

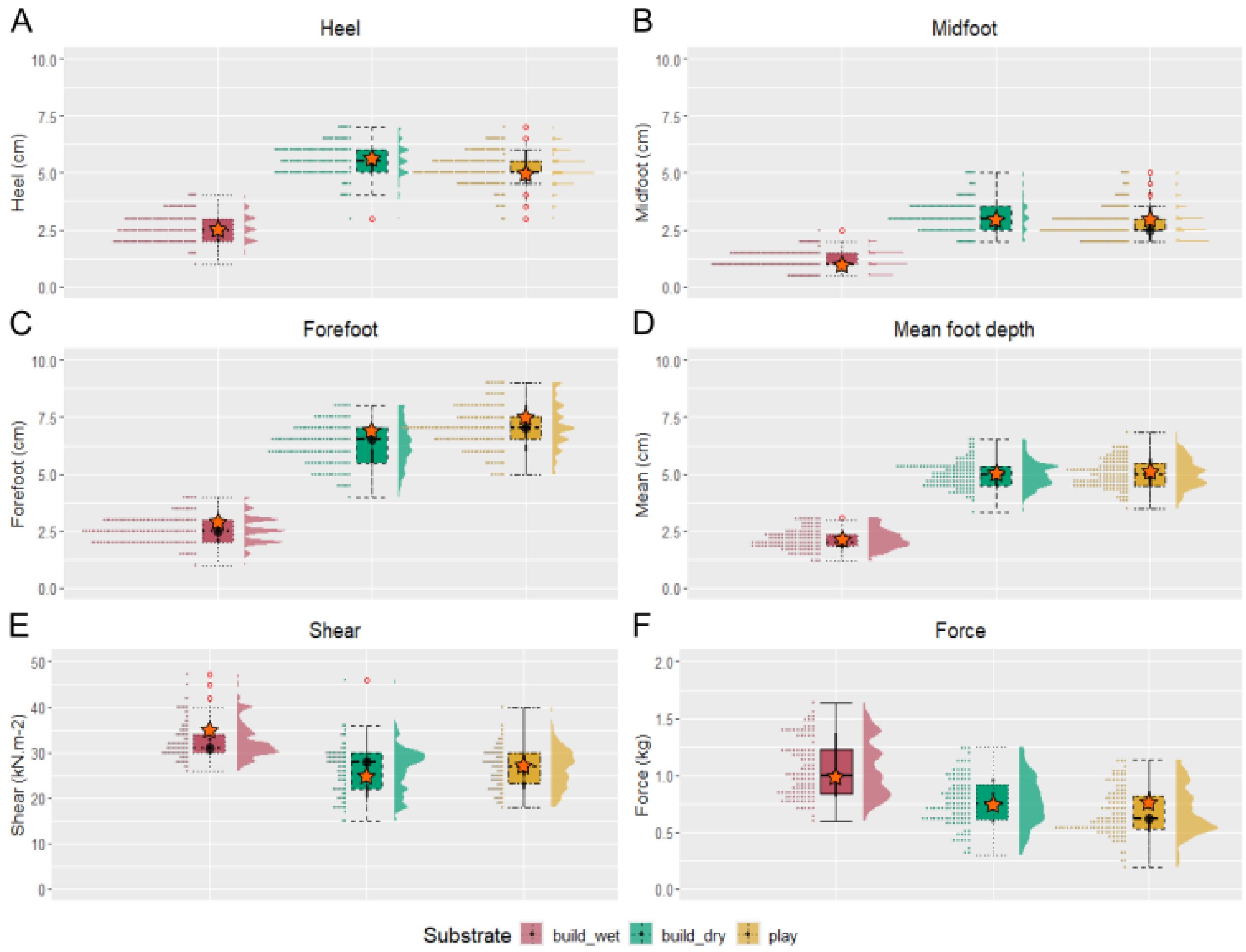

Fig. S1. Distribution of the control measurements to assess sand consistency taken prior to data collection for all participants (n=21). Measurements taken from the lead investigator footprints after walkways were modified at the (A) Heel, (B) Midfoot, (C) Forefoot, (D) Mean foot depth and measurements taken using the (E) shear vane and (F) Force gauge on the three sands: build wet sand (red), build dry sand (green) and play sand (yellow). Orange stars indicates the mean value of the control footprints (objective values). Red circles denotes outliers. The mean foot depth values were used as priority to assess sand consistency.

Journal of Experimental Biology • Supplementary information

Table S1. The results of the Tukey's HSD post-hoc analysis on the foot sinking depth measurements while walking on the three sand substrates. ANOVA found a significant ($p<0.001$) difference between substrates. These values were calculated using the lowest z-value positions for every stride for all participants combined (n=21) while walking on the sand: build wet sand, build dry sand and play sand: Values include both right and left (A) Calcaneus (n=1129), (B) Hallux (n=1129). Values used as a proxy for footprint depth.

A

Calcaneus

```
Multiple Comparisons of Means: Tukey Contrasts


Fit: aov(formula = CALC ~ Substrate, data = data_5)

Linear Hypotheses:
                           Estimate Std. Error t value Pr(>|t|)
build_dry - build_wet == 0  0.60181    0.07270   8.278   <1e-10 ***
play - build_wet == 0       2.00963    0.07013  28.655   <1e-10 ***
play - build_dry == 0       1.40782    0.06516  21.605   <1e-10 ***
---
Signif. codes:  0 '***' 0.001 '**' 0.01 '*' 0.05 '.' 0.1 ' ' 1
(Adjusted p values reported -- single-step method)
```

B

Hallux

```
Multiple Comparisons of Means: Tukey Contrasts


Fit: aov(formula = Hallux ~ Substrate, data = data_6)

Linear Hypotheses:
                           Estimate Std. Error t value Pr(>|t|)
build_dry - build_wet == 0  0.82802    0.09371   8.836   <2e-16 ***
play - build_wet == 0       1.80100    0.09041  19.921   <2e-16 ***
play - build_dry == 0       0.97298    0.08400  11.583   <2e-16 ***
---
Signif. codes:  0 '***' 0.001 '**' 0.01 '*' 0.05 '.' 0.1 ' ' 1
(Adjusted p values reported -- single-step method)
```

Journal of Experimental Biology • Supplementary information

Table S2. The results of the linear mixed-effect models on the spatiotemporal parameters: speed (ms^{-1}), stride length (m), stride width (m) and cycle time (s); fixed effects = substrate, speed and sex and random effects = participants. Statistical significance is set as $p<0.05$ with significant p-values shown in bold.

	Speed			Stride_Length			Stride_Width			Cycle_Time			Stance_Time			Swing_Time			Double_Limb_Support_Time			Duty_Factor		
Predictors	*Estimates*	*CI*	*p*	*Estimates*	*CI*	*p*	*Estimates*	*CI*	*p*	*Estimates*	*CI*	*p*	*Estimates*	*CI*	*p*	*Estimates*	*CI*	*p*	*Estimates*	*CI*	*p*	*Estimates*	*CI*	*p*
(Intercept)	1.22	1.16 – 1.27	**<0.001**	0.83	0.69 – 0.97	**<0.001**	0.17	0.10 – 0.23	**<0.001**	1.87	1.76 – 1.98	**<0.001**	1.37	1.27 – 1.46	**<0.001**	0.54	0.48 – 0.61	**<0.001**	0.79	0.70 – 0.88	**<0.001**	0.72	0.64 – 0.80	**<0.001**
Substrate [build_wet]	0.06	0.03 – 0.08	**<0.001**	-0.35	-0.52 – -0.17	**<0.001**	-0.01	-0.10 – 0.08	0.833	-0.34	-0.48 – -0.20	**<0.001**	-0.36	-0.49 – -0.23	**<0.001**	-0.02	-0.11 – 0.06	0.628	-0.27	-0.37 – -0.16	**<0.001**	-0.06	-0.16 – 0.05	0.292
Substrate [Floor]	0.15	0.13 – 0.17	**<0.001**	-0.04	-0.21 – 0.13	0.630	-0.07	-0.15 – 0.02	0.112	-0.25	-0.39 – -0.12	**<0.001**	-0.25	-0.37 – -0.13	**<0.001**	-0.03	-0.11 – 0.06	0.546	-0.14	-0.26 – -0.03	**0.015**	-0.08	-0.18 – 0.02	0.110
Substrate [play]	-0.03	-0.05 – -0.01	**0.001**	0.05	-0.11 – 0.20	0.553	-0.05	-0.13 – 0.03	0.192	0.08	-0.04 – 0.20	0.210	-0.09	-0.20 – 0.02	0.111	0.05	-0.03 – 0.13	0.198	-0.05	-0.15 – 0.05	0.316	-0.10	-0.19 – -0.01	**0.034**
Sex [M]	0.08	-0.01 – 0.16	0.080	-0.30	-0.51 – -0.09	**0.005**	-0.04	-0.14 – 0.06	0.483	-0.20	-0.37 – -0.04	**0.017**	-0.23	-0.37 – -0.08	**0.003**	-0.01	-0.11 – 0.09	0.823	-0.12	-0.25 – 0.01	0.073	-0.15	-0.27 – -0.03	**0.017**
Substrate [build_wet] × Sex [M]	0.01	-0.03 – 0.04	0.600	0.34	0.07 – 0.61	**0.015**	-0.12	-0.25 – 0.02	0.083	0.21	-0.00 – 0.43	0.054	0.34	0.15 – 0.54	**0.001**	-0.10	-0.23 – 0.03	0.147	0.27	0.11 – 0.43	**0.001**	0.21	0.05 – 0.38	**0.011**
Substrate [Floor] × Sex [M]	-0.01	-0.04 – 0.02	0.501	-0.06	-0.30 – 0.17	0.600	0.00	-0.11 – 0.12	0.989	0.08	-0.10 – 0.27	0.395	0.12	-0.04 – 0.29	0.143	-0.02	-0.14 – 0.10	0.772	0.05	-0.11 – 0.21	0.548	0.13	-0.01 – 0.27	0.075
Substrate [play] × Sex [M]	-0.03	-0.06 – -0.00	**0.023**	-0.05	-0.27 – 0.17	0.663	0.06	-0.05 – 0.17	0.263	0.06	-0.12 – 0.23	0.535	0.19	0.02 – 0.35	**0.024**	-0.01	-0.13 – 0.10	0.797	0.08	-0.06 – 0.23	0.255	0.26	0.13 – 0.40	**<0.001**
Speed				0.48	0.37 – 0.60	**<0.001**	-0.04	-0.09 – 0.02	0.207	-0.58	-0.67 – -0.49	**<0.001**	-0.51	-0.59 – -0.44	**<0.001**	-0.10	-0.15 – -0.04	**0.001**	-0.38	-0.45 – -0.31	**<0.001**	-0.09	-0.16 – -0.03	**0.005**
Substrate [build_wet] × Speed				0.25	0.11 – 0.40	**0.001**	0.01	-0.06 – 0.08	0.817	0.25	0.14 – 0.37	**<0.001**	0.27	0.17 – 0.38	**<0.001**	0.01	-0.06 – 0.08	0.694	0.20	0.11 – 0.29	**<0.001**	0.05	-0.03 – 0.14	0.227
Substrate [Floor] × Speed				-0.03	-0.16 – 0.11	0.685	0.04	-0.02 – 0.11	0.217	0.15	0.04 – 0.26	**0.006**	0.17	0.08 – 0.27	**<0.001**	0.00	-0.07 – 0.07	0.977	0.10	0.01 – 0.19	**0.028**	0.08	-0.00 – 0.16	0.061
Substrate [play] × Speed				-0.04	-0.17 – 0.08	0.500	0.04	-0.02 – 0.11	0.172	-0.07	-0.17 – 0.03	0.173	0.07	-0.02 – 0.17	0.127	-0.05	-0.11 – 0.02	0.147	0.05	-0.04 – 0.13	0.292	0.07	-0.00 – 0.15	0.059
Speed × Sex [M]				0.31	0.15 – 0.47	**<0.001**	0.02	-0.06 – 0.10	0.586	0.22	0.09 – 0.35	**0.001**	0.22	0.11 – 0.33	**<0.001**	0.03	-0.05 – 0.11	0.426	0.11	0.01 – 0.21	**0.032**	0.11	0.02 – 0.21	**0.019**
(Substrate [build_wet] × Speed) × Sex [M]				-0.28	-0.50 – -0.07	**0.008**	0.08	-0.02 – 0.19	0.122	-0.18	-0.35 – -0.02	**0.031**	-0.28	-0.43 – -0.13	**<0.001**	0.06	-0.04 – 0.16	0.226	-0.21	-0.34 – -0.09	**0.001**	-0.17	-0.29 – -0.04	**0.010**
(Substrate [Floor] × Speed) × Sex [M]				-0.00	-0.18 – 0.18	0.965	0.00	-0.08 – 0.09	0.918	-0.09	-0.24 – 0.05	0.195	-0.12	-0.25 – 0.00	0.059	0.00	-0.09 – 0.09	0.918	-0.05	-0.17 – 0.07	0.436	-0.11	-0.21 – 0.00	0.053
(Substrate [play] × Speed) × Sex [M]				0.05	-0.13 – 0.23	0.596	-0.05	-0.14 – 0.04	0.261	-0.04	-0.18 – 0.11	0.619	-0.15	-0.28 – -0.02	**0.025**	0.02	-0.07 – 0.11	0.685	-0.07	-0.19 – 0.05	0.232	-0.21	-0.32 – -0.10	**<0.001**
**Random Effects**																								
σ^2	0.01			0.00			0.00			0.00			0.00			0.00			0.00			0.00		
τ_{00}	0.01 Participant			0.00 Participant			0.00 Participant			0.00 Participant			0.00 Participant			0.00 Participant			0.00 Participant			0.00 Participant		
ICC	0.60			0.64			0.46			0.66			0.65			0.47			0.58			0.28		
N	21 Participant			21 Participant			21 Participant			21 Participant			21 Participant			21 Participant			21 Participant			21 Participant		
Observations	933			922			922			931			487			787			301			931		
Marginal R^2 / Conditional R^2	0.316 / 0.727			0.610 / 0.861			0.060 / 0.493			0.617 / 0.868			0.672 / 0.884			0.345 / 0.651			0.691 / 0.871			0.075 / 0.336		

Journal of Experimental Biology • Supplementary information

Table S3. The results of the linear mixed-effect models on the mass normalised mechanical energy exchange variables: the recovery of mechanical energy (expressed as a percentage; R), relative amplitude (RA) and congruity (the time when potential energy and kinetic energy are moving in the same direction; CO). Fixed effects = substrate, speed and sex and random effects = participants. Statistical significance is set as p<0.05 with significant p-values shown in bold.

	R			RA			CO		
Predictors	*Estimates*	*CI*	*p*	*Estimates*	*CI*	*p*	*Estimates*	*CI*	*p*
(Intercept)	86.84	62.53 – 111.14	**<0.001**	0.98	0.05 – 1.92	**0.039**	29.07	2.41 – 55.74	**0.033**
Substrate [build_wet]	-17.98	-46.77 – 10.82	0.220	-0.15	-1.26 – 0.96	0.790	-7.12	-38.64 – 24.41	0.657
Substrate [Floor]	-13.42	-41.84 – 14.99	0.353	0.78	-0.32 – 1.88	0.162	15.73	-15.36 – 46.83	0.320
Substrate [play]	-17.79	-44.22 – 8.64	0.186	0.90	-0.13 – 1.92	0.086	-9.26	-38.19 – 19.66	0.529
Speed	-24.70	-45.09 – -4.31	**0.018**	-0.12	-0.91 – 0.66	0.757	-11.60	-33.95 – 10.74	0.307
Sex [M]	-0.67	-33.29 – 31.95	0.968	0.52	-0.73 – 1.77	0.411	-28.00	-63.82 – 7.81	0.125
Substrate [build_wet] × Speed	14.83	-9.35 – 39.02	0.228	0.16	-0.78 – 1.09	0.742	9.75	-16.73 – 36.24	0.469
Substrate [Floor] × Speed	12.57	-10.32 – 35.46	0.280	-0.58	-1.47 – 0.30	0.195	-6.75	-31.80 – 18.31	0.596
Substrate [play] × Speed	16.31	-5.97 – 38.59	0.151	-0.72	-1.58 – 0.14	0.100	7.30	-17.08 – 31.68	0.556
Substrate [build_wet] × Sex [M]	-21.22	-63.57 – 21.13	0.325	0.33	-1.31 – 1.96	0.695	44.03	-2.32 – 90.38	0.063
Substrate [Floor] × Sex [M]	-19.51	-58.27 – 19.25	0.322	-1.56	-3.06 – -0.06	**0.041**	18.97	-23.44 – 61.38	0.379
Substrate [play] × Sex [M]	2.42	-34.56 – 39.41	0.897	-0.92	-2.36 – 0.51	0.204	25.84	-14.62 – 66.30	0.210
Speed × Sex [M]	5.60	-20.55 – 31.76	0.673	-0.37	-1.37 – 0.64	0.474	21.65	-7.03 – 50.33	0.138
(Substrate [build_wet] × Speed) × Sex [M]	14.95	-18.57 – 48.47	0.381	-0.25	-1.54 – 1.04	0.704	-36.03	-72.72 – 0.65	0.054
(Substrate [Floor] × Speed) × Sex [M]	11.70	-18.17 – 41.58	0.441	1.08	-0.08 – 2.23	0.067	-17.68	-50.37 – 15.01	0.288
(Substrate [play] × Speed) × Sex [M]	-5.95	-35.95 – 24.05	0.696	0.67	-0.49 – 1.83	0.258	-20.49	-53.31 – 12.33	0.220
**Random Effects**									
σ^2	14.42			0.02			17.24		
τ_{00}	11.34 Participant			0.01 Participant			17.10 Participant		
ICC	0.44			0.31			0.50		
N	19 Participant			19 Participant			19 Participant		
Observations	269			269			269		
Marginal R^2 / Conditional R^2	0.136 / 0.517			0.143 / 0.407			0.150 / 0.573		

Journal of Experimental Biology • Supplementary information

Table S4. The results of the linear mixed-effect models on the maximum range of motion at the ankle, knee and hip in the sagittal plane for all subjects combined (n=21). Fixed effects = substrate, speed and sex and random effects = participants. Statistical significance is set as p<0.05 with significant p-values shown in bold.

	Ankle_ROM			Knee_ROM			Hip_ROM		
Predictors	*Estimates*	*CI*	*p*	*Estimates*	*CI*	*p*	*Estimates*	*CI*	*p*
(Intercept)	30.64	12.45 – 48.82	**0.001**	74.30	63.27 – 85.34	**<0.001**	74.68	62.73 – 86.63	**<0.001**
Substrate [build_wet]	23.86	3.96 – 43.76	**0.019**	-13.59	-25.60 – -1.59	**0.027**	-12.81	-25.88 – 0.27	0.055
Substrate [Floor]	35.66	8.23 – 63.09	**0.011**	11.54	-5.01 – 28.09	0.171	11.15	-6.88 – 29.17	0.225
Substrate [play]	11.96	-10.61 – 34.52	0.298	1.56	-12.06 – 15.17	0.822	0.89	-13.93 – 15.72	0.906
Speed	13.09	-1.49 – 27.68	0.078	1.82	-7.02 – 10.65	0.686	1.61	-7.97 – 11.19	0.741
Sex [M]	5.45	-27.22 – 38.11	0.743	-13.16	-32.93 – 6.61	0.191	-14.41	-35.87 – 7.06	0.188
Substrate [build_wet] × Speed	-21.44	-37.19 – -5.69	**0.008**	9.81	0.31 – 19.32	**0.043**	9.18	-1.18 – 19.53	0.082
Substrate [Floor] × Speed	-35.55	-56.21 – -14.90	**0.001**	-14.19	-26.65 – -1.73	**0.026**	-14.03	-27.61 – -0.46	**0.043**
Substrate [play] × Speed	-9.39	-28.08 – 9.30	0.324	-1.10	-12.37 – 10.18	0.848	-0.56	-12.84 – 11.72	0.928
Substrate [build_wet] × Sex [M]	-39.79	-79.09 – -0.50	**0.047**	24.54	0.83 – 48.25	**0.043**	28.33	2.51 – 54.15	**0.032**
Substrate [Floor] × Sex [M]	-52.80	-94.43 – -11.18	**0.013**	-26.65	-51.76 – -1.53	**0.038**	-25.81	-53.16 – 1.54	0.064
Substrate [play] × Sex [M]	-8.29	-46.31 – 29.73	0.668	23.00	0.05 – 45.94	**0.049**	25.08	0.10 – 50.07	**0.049**
Speed × Sex [M]	-5.86	-30.88 – 19.16	0.645	13.66	-1.47 – 28.79	0.077	14.55	-1.89 – 30.99	0.083
(Substrate [build_wet] × Speed) × Sex [M]	29.42	-0.27 – 59.11	0.052	-19.77	-37.68 – -1.85	**0.031**	-22.44	-41.95 – -2.93	**0.024**
(Substrate [Floor] × Speed) × Sex [M]	38.37	7.50 – 69.23	**0.015**	15.82	-2.80 – 34.44	0.096	15.27	-5.00 – 35.55	0.139
(Substrate [play] × Speed) × Sex [M]	8.59	-21.47 – 38.65	0.574	-17.74	-35.88 – 0.40	0.055	-19.45	-39.20 – 0.30	0.054
**Random Effects**									
σ^2	31.25			11.37			13.49		
τ_{00}	26.46 Participant			10.93 Participant			11.43 Participant		
ICC	0.46			0.49			0.46		
N	21 Participant			21 Participant			21 Participant		
Observations	379			379			379		
Marginal R^2 / Conditional R^2	0.277 / 0.609			0.425 / 0.707			0.400 / 0.675		

Journal of Experimental Biology • Supplementary information

Table S5. The results of the linear mixed-effect models on the integrated EMG data for the muscles BFL, RF, VL, VM, TA, MG, LG and SOL; fixed effects = substrate, speed and sex and random effects = participants. Statistical significance is set as p<0.05 with significant p-values shown in bold.

	BFL			RF			VL			VM			TA			MG			LG			SOL		
Predictors	*Estimates*	*CI*	*p*	*Estimates*	*CI*	*p*	*Estimates*	*CI*	*p*	*Estimates*	*CI*	*p*	*Estimates*	*CI*	*p*	*Estimates*	*CI*	*p*	*Estimates*	*CI*	*p*	*Estimates*	*CI*	*p*
(Intercept)	45018.92	25458.55 – 64579.29	**<0.001**	45369.18	10591.15 – 80147.21	**0.011**	34398.22	-5982.36 – 74778.80	0.095	19374.44	-24615.36 – 63364.24	0.387	40152.53	3164.43 – 77140.63	**0.033**	45733.33	24507.63 – 66959.04	**<0.001**	39184.84	18000.90 – 60368.78	**<0.001**	21947.58	-6392.44 – 50287.60	0.129
Substrate [Build_wet]	-10593.45	-35108.42 – 13921.53	0.396	15318.05	-29004.32 – 59640.42	0.498	19958.65	-30621.15 – 70538.46	0.439	47315.02	-9133.76 – 103763.80	0.100	-13876.43	-61684.15 – 33931.28	0.569	2296.20	-24883.80 – 29476.19	0.868	3006.29	-23822.63 – 29835.22	0.826	13462.43	-22212.16 – 49137.03	0.459
Substrate [Floor]	4045.37	-21556.37 – 29647.11	0.756	59274.33	13019.78 – 105528.88	**0.012**	-18483.86	-71306.69 – 34338.97	0.492	-17386.75	-76261.32 – 41487.82	0.562	-44310.82	-94119.92 – 5498.27	0.081	12178.41	-16175.69 – 40532.50	0.399	-49865.55	-77873.74 – -21857.36	**0.001**	11549.86	-25701.48 – 48801.19	0.543
Substrate [Play]	-4507.82	-27384.12 – 18368.48	0.699	-13496.40	-54848.93 – 27856.13	0.522	38697.28	-8501.75 – 85896.32	0.108	67206.40	14547.71 – 119865.09	**0.012**	5997.87	-38588.20 – 50583.93	0.792	1427.10	-23929.32 – 26783.51	0.912	293.89	-24739.47 – 25327.26	0.982	27584.54	-5704.39 – 60873.48	0.104
Sex [M]	-21804.47	-51191.37 – 7582.43	0.146	-15899.34	-68179.96 – 36381.27	0.551	90599.51	29934.60 – 151264.43	**0.003**	71309.50	5178.73 – 137440.26	**0.035**	-25764.61	-81355.26 – 29826.03	0.363	-8145.71	-40055.10 – 23763.68	0.616	-36201.58	-68041.31 – -4361.85	**0.026**	4135.81	-38449.68 – 46721.31	0.849
Speed	-367.31	-15981.81 – 15247.19	0.963	2147.18	-26049.34 – 30343.69	0.881	5853.88	-26363.10 – 38070.85	0.721	22034.48	-13840.21 – 57909.17	0.228	-2054.71	-32383.27 – 28273.86	0.894	-3435.32	-20715.22 – 13844.58	0.696	1732.26	-15345.55 – 18810.07	0.842	18980.53	-3736.98 – 41698.05	0.101
Substrate [Build_wet] × Sex [M]	3415.22	-32737.15 – 39567.58	0.853	-14206.88	-79563.09 – 51149.34	0.670	-36707.14	-111297.60 – 37883.32	0.334	-89246.70	-172477.40 – -6016.01	**0.036**	16094.92	-54384.80 – 86574.64	0.654	-22516.95	-62593.56 – 17559.65	0.270	12656.74	-26906.09 – 52219.58	0.530	-32302.99	-84911.60 – 20305.63	0.228
Substrate [Floor] × Sex [M]	-7704.47	-42181.33 – 26772.40	0.661	-61044.31	-123348.81 – 1260.18	0.055	-74108.83	-145242.89 – -2974.77	**0.041**	-99381.37	-178701.42 – -20061.32	**0.014**	54905.82	-12225.68 – 122037.33	0.109	-31472.82	-69670.62 – 6724.99	0.106	58839.36	21117.12 – 96561.61	**0.002**	-38340.89	-88508.02 – 11826.24	0.134
Substrate [Play] × Sex [M]	-2362.77	-35501.17 – 30775.63	0.889	3169.33	-56740.32 – 63078.98	0.917	-46408.77	-114780.69 – 21963.16	0.183	-107554.01	-183850.78 – -31257.25	**0.006**	24986.20	-39625.27 – 89597.66	0.448	-9789.21	-46526.65 – 26948.24	0.601	8226.30	-28038.89 – 44491.48	0.656	-17874.64	-66097.67 – 30348.38	0.467
Substrate [Build_wet] × Speed	6315.89	-13718.52 – 26350.31	0.536	-13583.25	-49804.36 – 22637.85	0.462	-16020.80	-57356.23 – 25314.64	0.447	-39704.10	-85834.57 – 6426.37	0.091	13128.04	-25940.01 – 52196.09	0.510	-6043.71	-28255.56 – 16168.13	0.593	-4004.23	-25929.51 – 17921.05	0.720	-13143.87	-42298.20 – 16010.46	0.376
Substrate [Floor] × Speed	-4320.28	-24282.46 – 15641.90	0.671	-37215.74	-73282.27 – -1149.22	**0.043**	19677.88	-21509.10 – 60864.85	0.349	14929.45	-30978.41 – 60837.31	0.523	33024.97	-5815.53 – 71865.46	0.095	-10506.34	-32615.47 – 11602.80	0.351	38826.49	16987.66 – 60665.31	**0.001**	-15435.62	-44481.36 – 13610.11	0.297
Substrate [Play] × Speed	4410.52	-14636.07 – 23457.10	0.649	11508.87	-22920.91 – 45938.64	0.512	-31784.13	-71081.59 – 7513.32	0.113	-56178.32	-100021.58 – -12335.06	**0.012**	-4959.11	-42081.27 – 32163.04	0.793	-1232.59	-22344.15 – 19878.97	0.909	-110.66	-20953.21 – 20731.90	0.992	-23095.09	-50811.14 – 4620.96	0.102
Sex [M] × Speed	12373.85	-10050.49 – 34798.19	0.279	6180.92	-34295.77 – 46657.61	0.764	-75619.69	-121887.63 – -29351.75	**0.001**	-62918.72	-114399.84 – -11437.60	**0.017**	15465.71	-28030.48 – 58961.90	0.485	3591.92	-21208.25 – 28392.10	0.776	26186.35	1665.79 – 50706.92	**0.036**	-4867.38	-37490.03 – 27755.28	0.770
(Substrate [Build_wet] × Sex [M]) × Speed	-2282.81	-30445.83 – 25880.21	0.874	10960.87	-39951.97 – 61873.71	0.673	32527.14	-25579.50 – 90633.79	0.272	72573.22	7736.32 – 137410.12	**0.028**	-16500.22	-71403.72 – 38403.29	0.555	18523.53	-12696.28 – 49743.34	0.244	-11791.07	-42610.80 – 19028.66	0.453	24037.27	-16945.28 – 65019.82	0.250
(Substrate [Floor] × Sex [M]) × Speed	3402.34	-22915.79 – 29720.47	0.800	35827.75	-11731.09 – 83386.59	0.140	51943.11	-2357.55 – 106243.78	0.061	67304.62	6759.10 – 127850.13	**0.029**	-42146.61	-93385.86 – 9092.63	0.107	20177.56	-8979.38 – 49334.51	0.175	-51924.21	-80719.19 – -23129.22	**<0.001**	28166.37	-10128.78 – 66461.51	0.149
(Substrate [Play] × Sex [M]) × Speed	5057.23	-21501.21 – 31615.68	0.709	-3088.37	-51102.80 – 44926.07	0.900	35909.61	-18886.39 – 90705.61	0.199	85159.20	24010.90 – 146307.50	**0.006**	-18511.49	-70295.23 – 33272.25	0.483	9009.44	-20433.83 – 38452.71	0.548	-5275.83	-34340.34 – 23788.68	0.722	16328.70	-22319.23 – 54976.64	0.407
**Random Effects**																								
σ^2	37521482.98			122711914.37			159722716.83			199130430.29			142941764.23			46159320.80			44950815.96			79464947.49		
τ_{00}	72886593.08 Participant			138909434.46 Participant			314930010.47 Participant			165842570.44 Participant			80072319.18 Participant			42530085.97 Participant			64072190.55 Participant			136862658.98 Participant		
ICC	0.66			0.53			0.66			0.45			0.36			0.48			0.59			0.63		
N	19 Participant			19 Participant			19 Participant			19 Participant			19 Participant			19 Participant			19 Participant			19 Participant		
Observations	676			676			676			676			676			676			676			676		
Marginal R^2 / Conditional R^2	0.096 / 0.693			0.125 / 0.589			0.090 / 0.694			0.120 / 0.520			0.060 / 0.397			0.100 / 0.531			0.124 / 0.639			0.048 / 0.650		
